# USP10 deubiquitinates Tau, mediating its aggregation

**DOI:** 10.1038/s41419-022-05170-4

**Published:** 2022-08-20

**Authors:** Zhen Wei, Kuan Zeng, Jichang Hu, Xing Li, Fang Huang, Bin Zhang, Jian-Zhi Wang, Rong Liu, Hong-Lian Li, Xiaochuan Wang

**Affiliations:** 1 Department of Pathophysiology, School of Basic Medicine, Key Laboratory of Education Ministry/Hubei Province of China for Neurological Disorders, Tongji Medical College, Huazhong University of Science and Technology, Wuhan, China; 2 Co-innovation Center of Neuroregeneration, Nantong University, Nantong, JS China; 3 Department of Pathology and Pathophysiology, School of Medicine, Jianghan University, Wuhan, China; 4 Shenzhen Huazhong University of Science and Technology Research Institute, Shenzhen, China

**Keywords:** Molecular biology, Neurological disorders

## Abstract

Normal Tau promotes the assembly and stabilization of microtubules, thus, maintaining axon transport. In Alzheimer’s disease (AD), Tau aggregation causes it to lose these above-mentioned functions. However, the molecular mechanism leading to Tau aggregation in AD remains ambiguous. Here, we report that USP10, one of the important deubiquitinases (DUBs), is involved in Tau aggregation. We found that USP10 is upregulated in postmortem human AD and APP/PS1 mice brains, but not in P301S mice brains. Moreover, in primary neuronal cultures, Aβ_42_ induces a dose-dependent USP10 upregulation, an increase in the levels of both total and phosphorylated Tau, as well as a markedly elevated Tau binding with USP10, that is accompanied by a significantly decreased Tau ubiquitination. In addition, overexpression of USP10 directly causes an increase in the levels of total and phosphorylated Tau, induces Tau aggregation, and delays in Tau degradation. Results from mass spectrometry, reciprocal immunoprecipitation, and immunofluorescence assays strongly prove Tau’s interaction with USP10. This is further supported by the Tau307–326K and Tau341–378K peptides’ competitive inhibition of Tau binding with USP10, attenuating Tau hyperphosphorylation and Tau deubiquitination. Together, our data strongly indicate that USP10 plays a critical role in mediating Tau aggregation via downregulating its ubiquitination and thus slowing down Tau turnover. Inhibition of USP10-Tau interaction might be therapeutically useful in the management of AD and related tauopathies.

## Introduction

Tau is a microtubule-associated protein enriched in neurons that functions as a microtubules stabilizer and therefore promotes axonal growth. The intracellular hyperphosphorylated aggregates (neurofibrillary tangles (NFTs)) in addition to the aggregated amyloid-beta (Aβ) are the two main features of AD [1]. Because abnormal Tau aggregation is linked to the pathogenesis of many neurodegenerative diseases [2–5], there is escalating interest in understanding the mechanism of clearance and degradation of Tau aggregates for better management of related tauopathies.

Tau could be degraded by the ubiquitin-proteasome system (UPS) and autophagy-lysosome pathway (ALP) [6–8]. Current knowledge indicates that UPS is the primary route for the degradation of soluble Tau fractions from PHF, while ALP mainly deals with the large insoluble fractions which cannot be transported to and unfolded by the proteasome [9, 10]. Moreover, it has been shown that the lysosome system can also be activated by recognizing polyubiquitination signals, indicating collaboration between the two systems. With soluble hyperphosphorylated Tau forms considered as the toxic culprit [11, 12], which may be the primary influence driving AD, the therapeutic interventions focusing on UPS are the focal point of this work. Studies in animal models indicate that early impairment of UPS results in neuronal dysfunction and Tau alterations of AD [13]. In the later stage of AD, proteasome activity has been reported to be significantly decreased by up to 56% [14], and upregulating the activity of proteasome could attenuate the accumulation of Tau [15]. Aggregated Tau and Aβ are found to directly inhibit the activity of proteasomes, which is an indication that the function of UPS is affected in the AD brain.

Ubiquitination is a process whereby target molecules are covalently modified with ubiquitin (Ubi). It is a dynamic process that can be reversed by deubiquitinating enzymes known as deubiquitinases (DUBs) [16]. There are about 100 DUBs encoded by the human genome [17]. Among them, the ubiquitin-specific proteases (USPs) are the largest family of DUBs [18]. USP10 is a highly conserved deubiquitinating enzyme among over 50 members and is extensively involved in the initiation and progression of a broad spectrum of cancer types, DNA damage response, and signaling pathways [19–21]. However, the role of USP10 in the nervous system remains enigmatic. USP10 deubiquitinates and increases the stability of p53 [22], which is a transcription factor that is reported to be a tumor suppressor in cancer, and further evidence highlighted its involvement in AD via enhancing Tau hyperphosphorylation [23]. According to Deng’s study, USP10 interacts with AMPK to form a positive feedforward loop and amplifies the activity of the AMPK [24]. Interestingly, AMPK is a well-known neuronal Tau kinase [25]. Meanwhile, USP10 could combine with G3BP to promote the formation of stress granules (SG) and co-locate with hyperphosphorylated Tau [26], and is thought to recruit aggregation-prone proteins such as TDP-43 and Tau [27–29]. Based on these observations, it is tempting to postulate that USP10 is involved in tauopathy, but how it promotes AD pathogenesis remains unclear.

In this study, we have clarified that the level of USP10 is upregulated in AD patients and APP/PS1 transgenic mice. Our results showed that USP10 could be induced by Aβ_42_ oligomers, which have been previously reported to drive AD pathogenesis via complex unclear processes and trigger the spread of Tau pathology [30, 31]. We here showed that USP10 specifically removes ubiquitin from Tau, and promotes its stability and hyperphosphorylation. By competitively interfering with USP10-Tau interaction we found that it could be possible to increase Tau ubiquitination and therefore its clearance in Aβ-toxicity AD models. Therefore, blocking USP10-induced p-Tau accumulation could be an important therapeutic strategy in the early stage of AD.

## Results

### USP10 is upregulated in postmortem AD patients’ brains and APP/PS1 mice

**Fig. 1:**
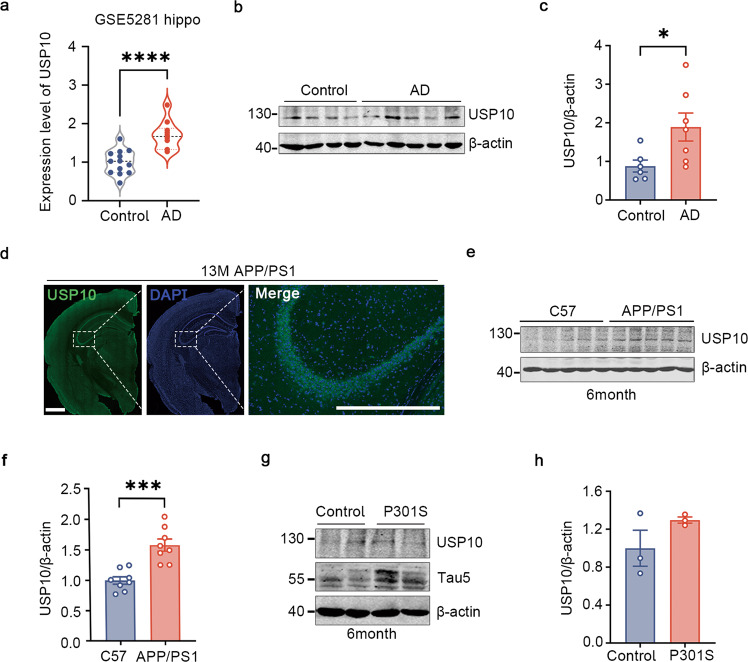
USP10 is significantly increased in AD. **a** Analysis of USP10 differentially gene expression level of GSE5281. **b** The level of USP10 in the postmortem hippocampus brain was measured by western blotting (WB). **c** Densitometry of WB shows a significant increase in AD compared to control. *n* = 6 control and 7 AD. **d** Immunofluorescence staining of USP10 in 13-month APP/PS1 mice. Scale bar = 500 μm. **e**, **f** The level of USP10 was significantly increased in the hippocampus of 6-month-old APP/PS1 mice, *n* = 8. **g**, **h** USP10 and Tau5 were detected in the hippocampus of 6-month-old P301S mice; *n* = 3. All data represent mean ± SEM, *p*-value significance is calculated from an unpaired two-tailed t-test. *P < 0.05, ***P < 0.001, ****P < 0.0001 vs control.

USP10 is believed to be involved in AD but the mechanism is unclear. To investigate that, we first evaluate its expression level in AD patients and animal models’ brains. GSE5281 dataset was used to evaluate the gene expression trend of USP10 in the postmortem. According to the array, USP10 presented a 1.6-fold upregulation in AD patients’ hippocampi compared with normal aged control (Fig. 1a). This result was confirmed by western blotting in postmortem frozen hippocampus tissue as the USP10 protein level was also found to be upregulated (Fig. 1b, c). Moreover, in APP/PS1 mice model of AD, IF showed prominent USP10 signals in the hippocampus (Fig. 1d). Hippocampi from 6- or 13-month-old APP/PS1 mice were examined and the results showed that USP10 level was higher in APP/PS1 mice compared to the controls (Fig. 1e, f and Supplementary Fig. 1). These mice are transgenic mouse models that overproduce Aβ, with the deposition of Aβ beginning at 3–4 months of age in the hippocampus. However, the level of USP10 exhibits no significant difference in 6-month-old P301S mice, a widely used tauopathy model (Fig. 1g, h). These results demonstrate that USP10 is upregulated in AD and might be associated with Aβ toxicity.

### Aβ_42_ triggers USP10 upregulation, inducing deubiquitination and hyperphosphorylation of Tau

**Fig. 2:**
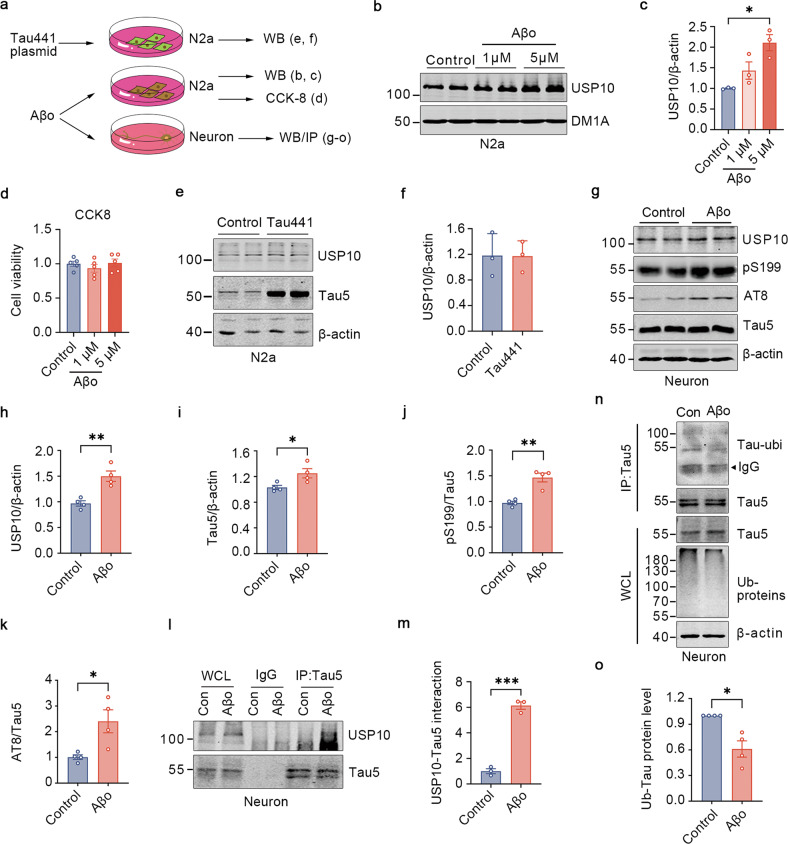
Aβo triggers USP10 upregulation inducing deubiquitination and hyperphosphorylation of Tau. **a** A Schematics of the experimental design. **b** Concentrations of 1 μM and 5 μM of Aβ_42_ oligomers(Aβo) were used after overnight incubation at 4 °C to treat N2a cells. **c** Quantification of WB bands showed that USP10 expression was increased by Aβo treatment; *n* = 3 independent experiments. **d** CCK-8 was used to detect the cell viability of N2a as indicated; *n* = 5 independent experiment. **e**, **f** Overexpression of the Tau441 plasmid did not affect the USP10 expression. *n* = 3 independent experiments **g**. Rat primary hippocampus neurons were grown until day 7, western blotting analyses for the level of USP10, Tau5, AT8, and pS199 under control conditions and 24 h after 2 μM Aβ_42_ oligomers treatment. **h**–**k**. Graphs showing the normalized protein levels of USP10, pS199, AT8(Ser202/Thr205), and Tau5; *n* = 4 independent experiments. **l** Aβo promotes the interaction of USP10 with Tau5. Rat primary neurons lysates were subjected to immunoprecipitation with control IgG, anti-Tau5 antibodies. **m** Graph showing the normalized level of USP10-Tau interaction. IP samples and whole-cell lysates (WCL) were analyzed using western blotting; *n* = 3 independent experiments. **n**, **o** Aβo triggered a decrease in Tau5 ubiquitination. Neuron lysates were incubated with protein A + G magnetic beads and lysates were immunoblotted as indicated; *n* = 4 independent experiments. All data represent mean ± SEM, *p*-value significance is calculated from a two-tailed t-test. *P < 0.05, **P < 0.01.

Being interested in dissecting the mechanism of USP10 upregulation in AD pathology, and observing that USP10 is upregulated in Aβ but not Tau model of AD, the following experiment was devised (Fig. 2a). We treated N2a cells with different concentrations of Aβ_42_ oligomers for 24 h. Immunoblotting results demonstrated that Aβ_42_ induced a dose-dependent augmentation in the USP10 level, with a significant effect at 5 μM (Fig. 2b, c). CCK8 assay showed there was no obvious toxicity for N2a as above treatment (Fig. 2d). Next, we overexpressed Tau in these cells, and consistent with the results in P301S mice, overexpression of Tau didn’t affect the level of USP10 (Fig. 2e, f). These results suggest that Aβ oligomers might be the trigger for the upregulation of USP10 in the AD setting.

According to the amyloid hypothesis, toxic Aβ accumulation in the brain is the dominant influence driving AD pathogenesis. Many studies have pointed out that human Aβ oligomers can induce hyperphosphorylation of Tau in cultured neurons [32]. We, therefore wondered whether USP10 might be involved in the effect of Aβ on Tau. To test this possibility, we incubated primary neurons with 2 μM of Aβ_42_ oligomers overnight. Compared to the control, the oligomers treatment produced a dramatic increase in the USP10 level (Fig. 2g, h). Interestingly, the concentrations of total Tau and phosphorylated Tau at Ser199 and Ser202/Thr205 (AT8) were dramatically elevated along with the increased USP10 expression (Fig. 2i–k). USP10 is one of the major members of the deubiquitinases family. Like ubiquitination, deubiquitination also requires protein to protein interaction. Therefore, to explore how USP10 affects both total and p-Tau levels we subsequently evaluate the interaction of Tau and USP10, and surprisingly this was found to be increased (Fig. 2l, m) and at the same time accompanied with a reduced ubiquitination level of Tau protein upon treatment with Aβ_42_ (Fig. 2n, o). Consistent with our findings in cultured neurons, USP10 was markedly increased, and the level of Tau5 and p-Tau were significantly increased in wild-type C57BL/6j mice injected with Aβ oligomers in the lateral ventricle for one week (Supplementary Fig. 2a–d). These results, together indicate that Aβ induces an upregulation of USP10 which might be responsible for the deubiquitination and buildup of both total and p-Tau proteins.

### USP10 induces Tau aggregation in primary neurons

**Fig. 3:**
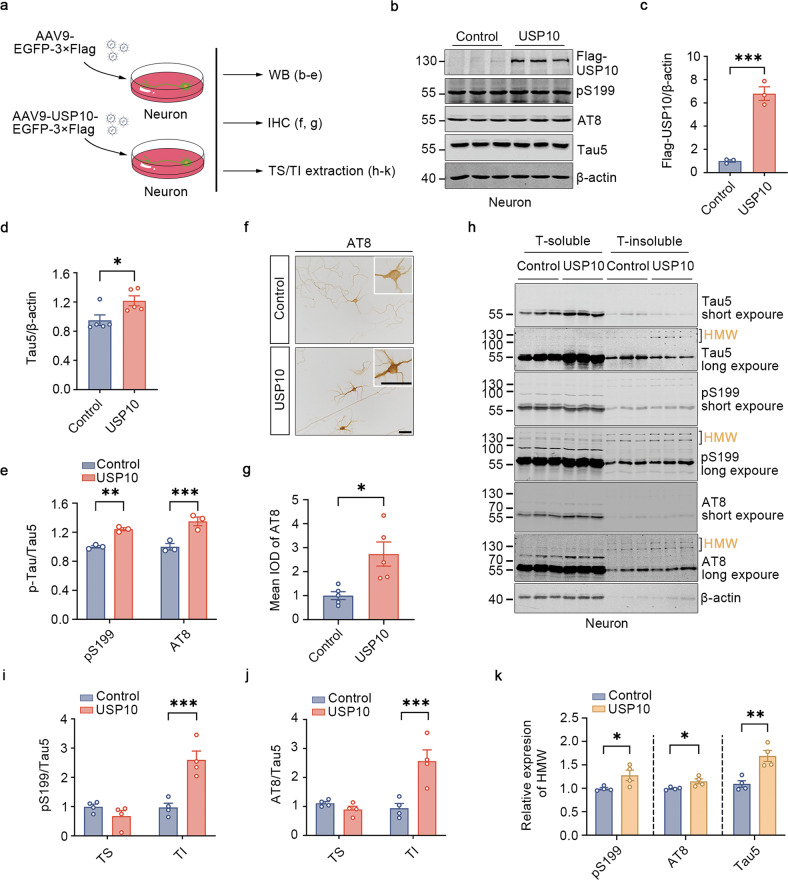
Overexpression of USP10 induces Tau aggregation in primary neurons. **a** A Schematics of the experimental design. **b** Primary neurons were infected with AAV9-FLAG-USP10 on DIV4, MOI = 10^5^, and 7 days later, samples were extracted and subjected to western blotting. **c**–**e** Graph showing normalized protein levels of Flag-USP10, pS199, AT8, and Tau5. **f** Immunocytochemistry showed the distribution of AT8 expressing USP10 compared to control. Scale bars, 50μm, inset also 50 μm. **g** Quantification of the mean of integrated optical density (Mean IOD), 3-10 cells per field of view (FOV) were counted, and five different FOV were taken in each group. **h** Neurons expressing USP10 were lysed, and 0.1% Triton-100 soluble and insoluble fractions were extracted as recommended by the protocol. WB results showed the level of Tau5, pS199, and AT8 (short exposure or long exposure). 100–130kd is considered to be the high molecular weight (HMW) of aggregated Tau proteins. **i**, **j** Graphs showing the normalized protein levels of AT8 and pS199 in T-soluble fractions and T-insoluble fractions under the aforementioned conditions. **k** HMW total Tau and p-Tau of insoluble fractions were quantified. Data are presented as mean ± SEM. *P < 0.05; **P < 0.01; ***P < 0.001.

We have demonstrated that Aβo could induce upregulation of USP10 in both cells and animals, and at the same time results in the buildup of Tau. To elucidate whether Tau is a downstream substrate of USP10, we employed primary neurons and carried western blotting, immunohistochemistry and triton-soluble and insoluble fraction extraction (Fig. 3a). Firstly, we overexpressed USP10 via adeno-associated virus (AAVs) in primary neurons. Western blotting results showed a substantial increase in Tau5 and phosphorylated Tau at Ser199 and Ser202/Thr205 (Fig. 3b–e). We also carried out an immunocytochemical assay and the results revealed a very strong AT8 staining in the USP10 overexpression group compared with control (Fig. 3f, g). In accordance with previously reported data, we also found that USP10 promotes the activity of AMPK in primary neuronal culture (Supplementary Fig. 3a–c). As expected, the addition of Aβ oligomers also increased the level of p-AMPK(T172) (Supplementary Fig. 3d–f).

Hyperphosphorylated Tau is prone to aggregation. To directly demonstrate the effect of USP10 on Tau accumulation, we extracted soluble and insoluble Tau fractions and separately examine the phosphorylation of Tau following USP10 overexpression. The immunoblotting showed that USP10 produced an increase in the soluble Tau5, while it specifically increased the insoluble phosphorylated Tau (Fig. 3h–j). Interestingly, USP10 overexpression led to the aggregation of insoluble Tau proteins at high molecular weights (100–130kD) (Fig. 3h, k). This suggests that USP10 upregulation alone is sufficient to induce Tau aggregation.

### USP10 deubiquitinates Tau and promotes Tau stability

**Fig. 4:**
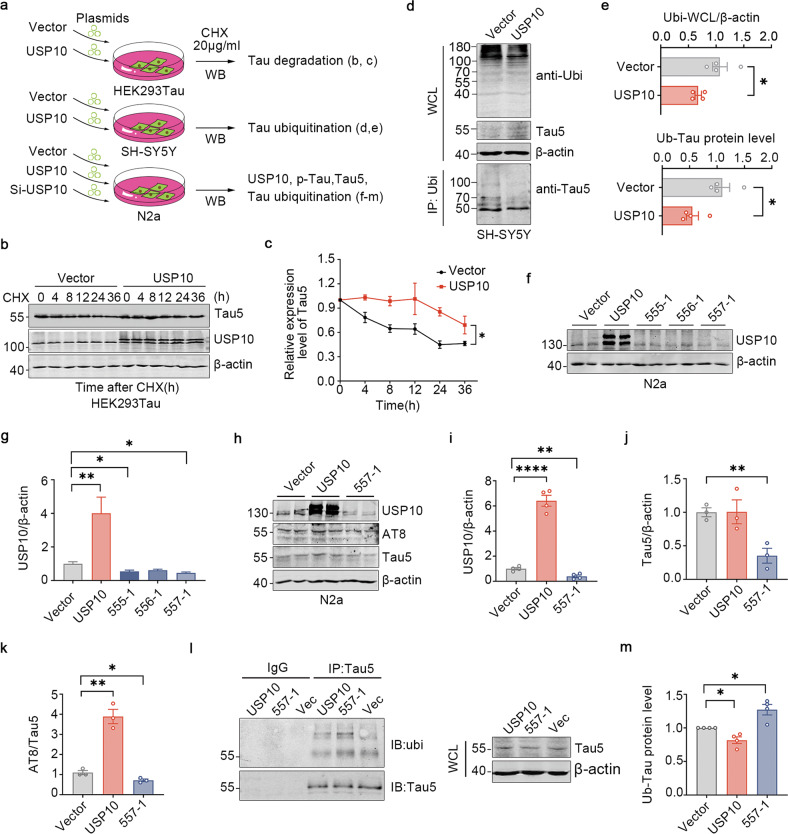
USP10 promotes Tau stability by slowing down its turnover. **a** A Schematics of the experimental design. **b** HEK293Tau cells were transfected with USP10 plasmids for 6 h, followed by treatment with cycloheximide (CHX, 20 μg/ml) for the indicated times to inhibit protein synthesis, and then analyzed by immunoblotting. **c** Quantification of the **b** blots, *n* = 3, *p*-value significance is calculated from a two-way ANOVA. **d** SH-SY5Y overexpressed with USP10 for 48 h, ubi was immunoprecipitated with anti-ubi polyclonal antibodies and then immunoblotted with monoclonal anti-Tau5 antibody. **e** Graph showing the normalized protein levels of ubiquitin of Tau and total protein; *n* = 4, *p*-value significance is calculated from a two-tailed t-test compared with vector. **f**, **g** N2a cells were transfected with the indicated siRNAs of USP10 for 48 h and the regulation of USP10 levels by the plasmids was evaluated. **h** N2a cells lysates expressing vector, USP10-OE, or USP10 siRNA plasmids were extracted. The panels show immunoblots of USP10, AT8, and Tau5. **i**–**k** Quantification of the relative USP10 and Tau5 protein levels are normalized to β-actin, relative AT8 level is normalized to Tau5. **l**, **m** USP10 regulated the ubiquitination of Tau as detected by IP in N2a cells. Anti-Tau5 immunoprecipates and WCL were analyzed using western blotting. *p*-value significance is calculated from a two-tailed t-test compared with vector, *P < 0.05, **P < 0.01, ****P < 0.0001. WCL, whole cells lysates.

Ubiquitination is a process that targets proteins for degradation, while deubiquitination decreases the degradation rate of affected proteins and thus might lead to the stability and build-up of target proteins. Since USP10 is a ubiquitin-specific protease, and we have found an increase in the total and p-Tau levels upon USP10 overexpression, it is possible that USP10 functions to stabilize Tau. In the current study, we employed HEK293Tau, SH-SY5Y and N2a cells to investigate the effect of USP10 on Tau ubiquitination and stability (Fig. 4a). Firstly, to prove that USP10 affects Tau stability per se, we treated control cells and cells overexpressing USP10 with cycloheximide (CHX), a protein synthesis inhibitor, and then measure the turnover of Tau. As expected, Tau stability was increased in USP10-infected primary neurons (Fig. 4b, c). It is likely that USP10 functions to deubiquitinate Tau to counteract the action of E3 ubiquitin ligases. Protein ubiquitination is essential for proteins to undergo ubiquitous proteasomal degradation systems. Indeed, as shown in Fig. 4d, the expression of USP10 not only decreased the level of ubiquitination of total proteins but also remarkably diminished Tau ubiquitination in the SH-SY5Y cell line, and effectively rescued Tau degradation (Fig. 4d, e, showed WCL-Tau5). To define the role of USP10 in regulating Tau levels, we depleted USP10 by using USP10-specific siRNA plasmids (Fig. 4f, g), and we found that knockdown of USP10 increased the ubiquitination of Tau and decreased the total Tau protein level as well as the sharp increase of AT8 (Fig. 4h–m). These results establish that Tau is a USP10 substrate, and USP10 negatively regulates Tau ubiquitination in cells leading to its accumulation.

### Molecular docking of USP10-Tau interaction interface

**Fig. 5:**
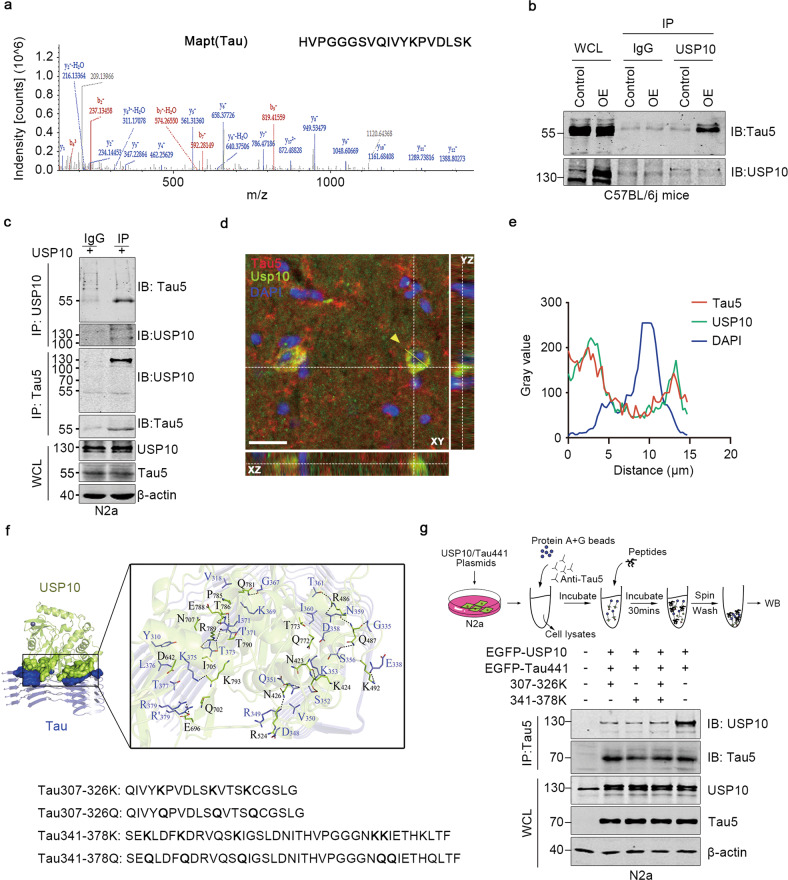
Synthesized peptides block USP10 interaction with Tau. **a** Mass spectrometry analysis of USP10-interacting proteins. The detected Mapt (Tau) typical mass spectrometry peptide spectra were listed. The list of USP10-interacting proteins is provided in Supplementary Table S3. **b** USP10 interacts with Tau5 in C57BL/6j mice tissue. AAV9-USP10 was injected into the hippocampus of wild-type C57BL/6j mice, tissues were immunoprecipitated with USP10 and subjected to western blotting. **c** USP10 interacts with Tau5 in N2a cells. Immunoblots of co-immunoprecipitation *(upper)* or reverse co-immunoprecipitation *(lower)* of USP10 and Tau5. WCL were analyzed using western blotting. **d** Confocal microscopy Z-stack imaging to hippocampus region of 13-month-old APP/PS1 mice. Immunostaining of Tau5 (Red), USP10 (Green), and DAPI (blue). X–Z, X-Y and Y-Z directions were shown. Yellow dotted lines represent the Z-depth of the 5 µm optical slice, and the yellow line represents the statistical area. The scale bar is 20 μm. **e** Statistical analysis of USP10, Tau5, and DAPI immunoreactivity. **f** Visualization of molecular docking of USP10 and Tau5 of the lowest energy, and the design of a series of blocking peptides targeting the USP10-Tau interaction interface. **g** Upper panel was a schematic of experimental process. N2a cells were co-transfected with USP10 and Tau. Cytoplasmic extracts were immunoprecipitated with a Tau5 antibody. The peptides Tau 307-326 K, Tau341-378K, or Tau 307-326 K + Tau341-378K at a concentration of 1 mM for 30 min at room temperature were used to compete with the USP10-Tau interaction in vitro. IP and WCL samples were blotted with the indicated antibodies.

To figure out the USP10-Tau interaction, Immunoprecipitation- mass spectrometry was performed. Firstly, the AAV9-USP10 virus was injected in the hippocampal CA3 region of 8-week-old C57BL/6j mice, and the whole-tissue lysate was immunoprecipitated using anti-USP10. Mapt (Tau) was identified (Fig. 5a). Reciprocal immunoprecipitation with anti-USP10 also showed Tau signal after pull-down in both mice tissue and N2a cells (Fig. 5b, c). Further, colocalization analysis using immunofluorescence staining of endogenous proteins showed colocalization of USP10 and Tau in 13-month-old APP/PS1 mice (Fig. 5d, e).

To further confirm the direct interaction between USP10 and Tau, we decided to carry out a USP10 competitive binding study by producing competitive USP10-binding Tau fragments. To generate a peptide capable of blocking the direct regulatory effect of USP10 on Tau, the precise mapping of the USP10-Tau binding interface is a prerequisite. Thus, we firstly performed molecular docking studies, the results of which displayed that USP10 exhibited a high binding affinity for Tau (free docking energy −25.33 KJ/mol) (Fig. 5f). With this knowledge in mind, and combined with the previously reported ubiquitination sites of Tau [33], we designed a series of peptides targeting the possible interaction sites of USP10 and Tau (Fig. 5f).

An in vitro competition assay was performed in order to confirm whether these peptides target the USP10-Tau interaction sites. Lysates from N2a cells co-expressing USP10 and Tau441 were immunoprecipitated with anti-Tau5 antibody and were then competed using 1 mM of Tau307-326K, Tau341-378K, and Tau307-326K + Tau341-378K peptides in tubes (Fig. 5g). The level of USP10 detection was much lower after competition with both peptides (Fig. 5g). The data above suggest that Tau directly interacts with USP10.

### Peptides targeting USP10-Tau interaction attenuates the accumulation of Tau in cells

**Fig. 6:**
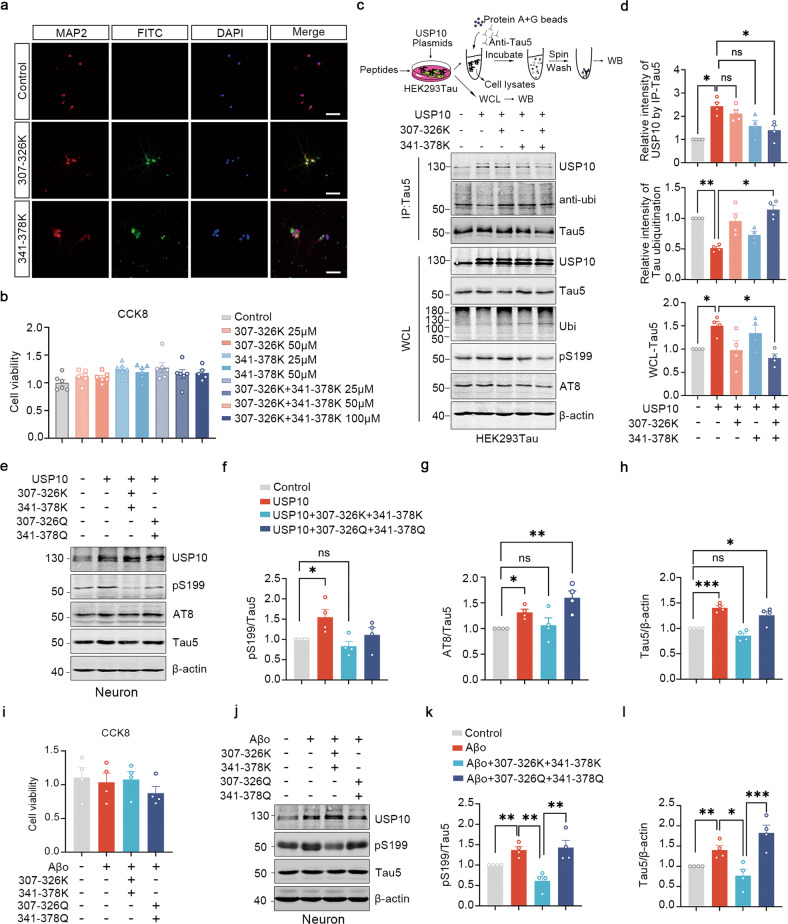
Interfering peptides attenuate both USP10 overexpression or Aβ-triggered Tau aggravation in primary cells. **a** IF micrographs of FITC (green), MAP2(red) and DAPI (blue) in the primary neurons incubated with 25 μM FITC-tagged Tau307-326K or FITC-tagged Tau341-378K for 24 h. The scale bar represents 100 μm. **b** The primary cells viability was evaluated with CCK8 by pretreating cells with different concentrations of peptides; *n* = 6 independent experiments. **c** Upper panel was a schematic of experimental process. HEK293Tau cells overexpressing full-length USP10 with the vehicle were pretreated with 50 μM of Tau 307-326 K, Tau341-378K, or Tau 307-326 K + Tau341-378K for 48 h. WB showed the effects of interfering peptides on USP10-Tau interaction, Tau ubiquitination, and the level of WCL’s USP10, Tau5, ubi, pS199, and β-actin. **d** Graphs showing the normalized USP10 and Tau ubiquitination level following immunoprecipitation with Tau5 antibody, and the WCL Tau5 level. **e**–**h** Rat primary neurons were infected with AAV9-USP10 for 5 days and co-incubated with 50 μM of Tau 307-326 K + Tau341-378K or Tau 307-326Q + Tau341-378Q for 48 h. Cells lysates were collected and analyzed by WB. Interfering peptides abrogated the USP10-OE induced p-Tau upregulation, while non-ubiquitination mimics failed. **i**–**l** Rat primary neurons preincubated with 50 μM Tau 307-326 K + Tau341-378K or Tau 307-326Q + Tau341-378Q for 12 h, then supplemented with 2 μM Aβ oligomers for 24 h. CCK-8 was used to detect the cell viability of primary neurons as indicated (**i**). Lysates were analyzed by WB (**j**). Interfering peptides abrogate the Aβ_42_ oligomers-induced upregulation of p-Tau at Ser199 (**k**) and Tau5 (**l)**, while non-ubiquitination mimics could not. All data are presented as mean ± SEM, *p*-value significance is calculated from one-way ANOVA, *P < 0.05; **P < 0.01; ***P < 0.001; ns not significant.

From the results shown so far, it is clear USP10 interaction with Tau plays a key role in the accumulation of Tau. Therefore, we thought that the interfering peptides would be functionally beneficial in attenuating tauopathy induced by USP10 overexpression or triggered by Aβ in primary cells. We firstly labeled these peptides with FITC to determine whether they can be successfully delivered into cells. Following overexpression of these peptides, a significant FITC signal was observed in fixed primary neurons, demonstrating that FITC-peptides were able to penetrate hippocampus neurons (Fig. 6a). We also performed a cell viability assay, and the synthesized peptides were found to exhibit scarce cytotoxicity at a concentration of up to 100 μM (Fig. 6b). The incubation of HEK293Tau cells with 50 μM of peptides for 24 h following transfection with USP10, inconsistent with in vitro studies, showed that treatment with both peptides together (Tau307-326K + Tau341-378K) is more effective than any single peptide treatment, as it induced a marked decrease in the USP10-Tau interaction, increased Tau ubiquitination, and at the same time decreased Tau5 level (Fig. 6c, d). Our results from Supplementary Fig. 3 showed increased activity of AMPK upon USP10 overexpression, so we assessed the activation of AMPK after peptides treatment. We found that the addition of peptides didn’t affect the activation of AMPK induced by USP10 (Supplementary Fig. 4).

Moreover, we infected primary neurons at DIV4 with AAV9-USP10 for 3 days and incubated them with 50 μM of Tau307-326K + Tau341-378K for 48 h. In line with the cell line experiment, western blotting results from lysates or primary neurons showed that the peptides reversed Tau hyperphosphorylation and decreased the level of total Tau (Fig. 6e–h). On the other hand, non-ubiquitination mimics mutant peptides, Tau307-326Q + Tau341-378Q, in which lysine residues (K) are substituted with glutamine (Q), lost their ability to reverse Tau upregulation (Fig. 6e–h).

The above results postulate that USP10 is involved in Tau pathology induced by Aβ. To explore the possible effect of interfering peptides in the Aβ toxicity setting, we incubated neurons with 50 μM of either Tau307-326K + Tau341-378K or Tau307-326Q + Tau341-378Q and then incubated them with Aβ_42_ oligomers for 24 h. CCK8 assay showed there was no obvious toxicity for primary neurons as above treatment (Fig. 6i). Western blotting results showed that the interfering peptides markedly decreased the pS199 and Tau5 induced by Aβ stimulation (Fig. 6j–l), however, the non-ubiquitination mimics (Tau307-326Q + Tau341-378Q) failed (Fig. 6j–l). We also extracted triton-soluble and triton-insoluble fractions and the results from western blotting demonstrated that the interfering peptides could reverse the Aβ-induced soluble toxic Tau culprits (Supplementary Fig. 5). Therefore, together, our results identify the role of the USP10-mediated amyloidogenic pathway in tauopathy.

## Discussion

Impaired intracellular protein degradation is known to be involved in AD [34], and abnormal protein aggregates are a hallmark in almost all neurodegenerative diseases. In the course of AD, small soluble Tau proteins that are produced at an early stage are considered to be highly toxic and are transformed into insoluble and relatively less toxic NFTs at the later stage of the disease. The proteasome activity is intensively inhibited as AD progresses, and highly hyperphosphorylated Tau proteins are observed in PHFs and this is partially responsible for the reduced Tau degradation. Ubiquitin is known to target proteins for degradation pathways [35, 36], and protein ubiquitination can be reversed by DUBs. As one of the important de-ubiquitinating enzymes, USP10 is reported to be involved in tauopathy but its role in AD is not well understood. In this research work, we found that USP10 is upregulated in both AD patients and APP/PS1 mice but not in 6-month-old P301S mice (Fig. 1). Moreover, Aβ stimulation increased the USP10 level in both the N2a cell line and primary neurons. We then subsequently mainly elaborate on the underlying mechanisms of how USP10 interacts with and deubiquitinates Tau, and promotes Tau hyperphosphorylation, resulting in its accumulation. Furthermore, interfering with USP10-Tau interaction reversed the tauopathy induced by USP10 overexpression and Aβ oligomers treatment. To our knowledge, this is the first study reporting USP10 as a deubiquitinase of Tau involved in Aβ-mediated toxicity in AD.

Aβ is a stressor considered as the primary influence driving AD. Since we found increased USP10 in both postmortem AD patients and APP/PS1 mice brains but not in P301S mice, we thought that Aβ might be a trigger of USP10. Our results from both cell and animal experiments showed an upregulation of USP10 upon Aβ stimulation. It has been reported that Aβ induced tauopathy through different pathways [37, 38]. Our results showed that total Tau, hyperphosphorylation of Tau, and especially the USP10-Tau interaction, were increased upon 2 μM Aβ stimulation (Fig. 2). The fact that the Aβ burden exists as early as 6 months of age in APP/PS1 mice suggests that USP10 may be involved in the Aβ amyloid hypothesis of p-Tau aggregation.

Phosphorylation of Tau preceded its aggregation. Here we demonstrated that USP10 increased soluble both total and phosphorylated Tau content, and this is consistent with reports from previous studies of DUBs [39–41]. Moreover, USP10 markedly promotes the soluble and insoluble phosphorylated Tau aggregation (Fig. 3). Hyperphosphorylated Tau is the main constituent of NFTs, one of the two main hallmarks of AD, and NFTs correlate with the progression of AD [42]. In the early stage of AD, smaller soluble Tau oligomers are considered to be highly toxic [43]. This suggests that at this early stage of AD, USP10 might trigger Tau phosphorylation and thus contributes at least in part to AD pathogenesis. Deng and colleagues reported that USP10 promoted AMPK activation, a well-known Tau kinase. In accordance with this, we found that overexpression of USP10 in primary neurons or Aβ stimulation of these neurons resulted in a significant increase in AMPK activity (Supplement Fig. 3). This may therefore be a possible mechanism by which USP10 promotes the Tau hyperphosphorylation, but further studies are needed to verify this hypothesis.

It is unclear how USP10 induces Tau aggregation. Moreover, a previous study has shown that USP10 does not interact with Tau but only promotes the formation of SGs to increase p-Tau-positive SGs aggregates in the AD [26]. However, we here reported conflicting results whereby USP10 was identified to interact with Tau leading to its deubiquitination (Figs. 4 and 5). There are about six different Tau isoforms from a single MAPT gene that are expressed in the human brain. To further confirm the USP10-Tau interaction, we performed IP-MS and co-IP assay by overexpressing the full-length Tau(2N4R) in mice or N2a cells, and also carry out immunofluorescence co-staining of USP10 and Tau in 13-month-old APP/PS1 mice, and all results revealed the USP10-Tau interaction (Fig. 5a–e). Taken together, these support our speculation of direct regulation of Tau by USP10. Indeed, data also showed a weak USP10-Tau interaction at normal physiological conditions in vivo and in vitro (Figs. 2l and 5b, control panels). However, USP10-Tau interaction was significantly enhanced under stressful conditions like Aβ toxicity.

Our data revealed a direct USP10-Tau interaction that might play a role in the USP10-mediated Tau pathology. Therefore, we thought that the Tauopathy might likely be blocked or at least attenuated by interfering with the USP10-Tau interaction. Recently, peptide therapeutics are gaining more and more significant importance in the clinical setting as a therapeutic means for the management of many diseases [44, 45]. We thus thought of constructing therapeutic peptides that might interfere with the USP10-Tau interaction. To this aim, we first carry out a molecular docking experiment to predict the mode of USP10-Tau interaction (Fig. 5f). The results from this experiment showed that Tau and USP10 binding region is U-shaped and divided into two parts. In addition, previous research reported the Tau ubiquitination sites in AD [33]. Thus, based on this and our docking experiment results, we designed and synthesized four peptides. Interestingly, Tau aggregation and hyperphosphorylation induced by USP10 overexpression and Aβ stimulation were found to be significantly abrogated by these peptides (Fig. 6). However, the activation of AMPK induced by USP10 does not seem to be influenced by these peptides (Supplementary Fig. 4). These results together are an indication that USP10 directly regulates p-Tau in addition to its indirect action. Whether our synthetic peptides could block or at least slow down the progression of AD in vivo still needs further studies.

Emerging evidence suggests that different polyUb linkages regulate a variety of cellular processes [46], and previous studies about USP10 reported different regulatory mechanisms of USP10 on different proteins. But, in the current study, we have not conducted in-depth studies on this matter, therefore, this needs further investigations. It should however be noted that our study found that USP10 is involved in the ubiquitination of both K63 and K48 linkage-specific ubiquitination sites (data not shown). This could explain why USP10 showed such diversity and thus, the detail is worth exploring.

Taken together, in this work we revealed a novel link between USP10 and AD which further enriches the currently available knowledge. In particular, we showed that USP10 is a Tau DUB that directly binds Tau, deubiquitinating it, and thereby promoting Tau aggregation. Our data are of significant importance due to the fact that early oligomeric forms of Tau are generally considered to be toxic culprits in AD and related tauopathies. We have also complemented the Aβ-mediated approach to Tau pathology involving USP10, and also opened new avenues for the development of small interfering peptides that could specifically target the accumulation of pathological forms of Tau in AD and related Tauopathies.

## Materials and methods

### Cell culture, mice, and human tissue samples

HEK293Tau cells were cultured in DMEM-high glucose medium (Gibco, Invitrogen; Bleiswijk, Netherlands) supplemented with 200 mg/mL G418 and 10% fetal bovine serum (Gibco BRL, Gaithersburg, MD, USA); SH-SY5Y and N2a cells were cultured also in DMEM-high glucose containing 10% fetal bovine serum. Primary rat hippocampal neurons were isolated from rat embryos (day-17 or 18) and maintained in neuroGro (Gibico, #21103049), supplement with 1 × B27 supplement (Gibico, #17504044) and 1×GlutaMAX and 100 U/ml penicillin-streptomycin (Gibico, #15140122). Neurons cultured were infected with AAVs at 4 days in vitro (DIV4). The cells were maintained at 37 °C in a humidified atmosphere containing 5% CO_2_. Pregnant Sprague Dawley rats were obtained from the Experimental Animal Center of Tongji Medical College, Huazhong University of Science and Technology. Male C57BL/6j, APP/PS1, and P301S mice were purchased from the Charles River laboratories. All mice were used at mentioned weeks of age and were housed under standard conditions 12 h light and 12 h dark with water and food ad libitum. Postmortem brain samples were dissected from frozen brains of 7 AD cases and 6 non-demented controls from the China Brain Bank in Zhejiang University School of Medicine. More detailed information is in Supplementary Table S1.

### Plasmids, viruses, chemicals, and antibodies

The plasmids encoding EGFP-USP10 (GV 230), EGFP-Vector, pIRES2-EGFP-Tau40, pIRES2-EGF, and viruses of AAV9-CMV bGlobin-USP10-EGFP-3FLAG-WPRE-hGH-polyA and AAV9-CMV bGlobin-EGFP-3FLAG-WPRE-hGH-polyA were constructed and packaged by Shanghai Genechem Co., Ltd. Aβ_1-42_ peptides were manufactured by Chinapeptides co. ltd, (>95% purify) and lyophilized powder was stored at −80 °C. When used, the powder was first dissolved in DMSO. The solution was diluted with medium and incubated overnight at 4 °C to prepare Aβ oligomers. Tau307-326K/Q, Tau341-378K/Q peptides were commercially synthesized with a purity of >95% (Bioyeargene Biosciences, Wuhan, China). The peptides were dissolved in 50 mM Tris-HCl (pH 7.4) to make a 2 mM stock solution and which was diluted with culture medium when used. Antibodies and reagents employed in this study are listed in Supplementary Table S2.

### Stereotaxic Injection

For in vivo AD mouse model, eight 8-week-old C57BL/6j mice were used. Mice were divided into two groups at random, and injected with Aβ_42_ oligomers(410 pmol/5 μl) in the lateral ventricle under anesthesia with isoflurane and maintaining in anesthesia with continuous low-flux ventilation. Bilateral ventricles stereotaxic injections (anteroposterior, −0.2 mm; mediolateral, ±0.9 mm; dorsoventral, −2.3 mm from bregma) of Aβ_42_ oligomers were performed with a Hamilton syringe at a rate of 0.2 µl/min, 2.5 μl each side. And the needle was kept in place for additional 2 min before withdrawal. Suture the incision and apply lincomycin lidocaine gel, and one week later, mice were sacrificed for western blotting.

### Molecular docking

Molecular docking between USP10 and Tau was performed using RosettaDock [47]. The crystal structure of Tau (PDB ID: 7NRS) and USP10 (PDB ID: 3N3K) were obtained from (http://www.rcsb.org) protein data bank. The lowest-energy docking solutions from the top 100 search results were chosen since they all showed relevant USP10-Tau interactions. This program was performed by Yangene biological technology (Wuhan) Co., LTD.

### Soluble and insoluble fraction extraction

For cell fractionation analysis, a general protocol that allowed for the separation of Triton-100 soluble and 1% SDS soluble fractions was used. Cells were washed 3 times in ice-cold PBS and lysed in 1% Triton lysis buffer (1%Triton-100(v/v), 150 nM NaCl, and 50 mM Tri-HCl, pH 7.6) containing cocktail and PMSF. The lysates were collected with a cell scraper and kept on ice for 15 min. The supernatant of samples (Triton-soluble fraction) were collected after being centrifuged at 16000 × *g* for 30 min at 4 °C. The pellet was resuspended in 1% SDS lysis buffer (1% SDS (w/v), 150 nM NaCl and 50 mM Tri-HCl, pH 7.6) containing cocktail and PMSF. Samples were ultrasonicated for 15 s to promote dissolution and centrifuged at 16000 × *g* for 30 min at room temperature (RT). Finally, the supernatant was collected (Triton-insoluble fraction). Protein concentration in the Triton-soluble fraction was quantified by Bicinchoninic acid (BCA) protein kit (Pierce, Rockford, IL, USA), a final concentration of β-mercaptoethanol (BME) and bromophenol blue (3:1) were added.

### Immunoprecipitation (IP)

Cells or brain tissues were lysed in RIPA lysis buffer (Weak) (50 mM Tris-HCl, pH 7.4, 1 mM EDTA, 150 mM NaCl, 1% NP-40, 1 mM EGTA, 2.5 mM sodium pyrophosphate, 1% sodium deoxycholate, 1 mM Na_3_VO_4_ and 1 mM β-glycerophosphate) (Beyotime, #P0013D) for 10 min at 4 °C. The supernatant was collected by centrifuging at 12000 rpm for 10 min at 4 °C. The protein concentration was diluted into 1 μg/μl for IP. The lysates were incubated with 2 μl primary antibodies overnight, then added 30 μl protein A + G magnetic beads for 4-6 hours. A magnetic rack was used to discard the supernatant and the beads were washed 3 times with RIPA buffer. A 30 μl 1×loading buffer was added and boiled for 10 min, then the supernatant was collected and analyzed by western blotting.

### Western blotting analysis

The procedure was as previously described [48]. Cells and brain tissues were lysed by RIPA buffer weak or strong respectively. After being centrifuged and boiled in SDS buffer for 10 min, proteins were collected. Proteins were separated by 10% SDS polyacrylamide gel electrophoresis and then transferred to a nitrocellulose membrane. After blocking in 5% nonfat milk for 30 min, the membranes were incubated with primary antibodies overnight at 4 °C. Finally, the membranes were incubated with the secondary antibody at room temperature for 1 h. Odyssey Infrared Imaging System was used to visualize immunoreactive bands. Bands intensity readings were obtained using ImageJ (Fiji) software.

### Immunofluorescence microscopy assay

The brains of mice were perfused with normal saline and 4% paraformaldehyde for immunofluorescence. Briefly, the brains were cut into 25 μm sections and rinsed in PBS. Slices were permeabilized with 0.5% Triton-PBS for 30 min and blocked with 3% BSA. After incubating the first primary antibody for 24 h another primary antibody was performed. Finally, the slices were incubated with Alexa Fluor® 594- or 488-conjugated secondary antibodies for 1 h at RT. Antifade mounting medium with DAPI was used, images were acquired through a fluorescence microscope (Zeiss LSM780) and co-localization was analyzed with Plot Profile plugin of ImageJ software.

### Immunocytochemistry

Cells plated on coverslips were fixed with 4% paraformaldehyde. After permeabilization with buffer containing 0.3% H_2_O_2_, 0.5% Triton-100, cells were blocked with 3% BSA and incubated with AT8 primary antibody overnight at 4 °C. Subsequently, after washing 3 times, anti-mouse secondary antibodies were used. Finally, cells were stained with DAB (Zsbio Business Shop, Beijing, China). Images of p-Tau (AT8)-positive cells in the field of view were digitized using a microscope (Nikon, Ni-E) equipped with ×20 objectives.

### Mass spectrometry analysis

Lysates of 200 μg were extracted from the hippocampus tissue of mice overexpressing USP10 using RIPA buffer (weak) with 1×proteinase inhibitor cocktail and 1 mM PMSF on ice. The protein concentration was diluted to 1 μg/μl. Samples were precleared from non-specific binding proteins by incubation with protein A + G magnetic beads for 30 min at 4 °C. The tubes were placed on a magnetic rack for 1 min and the supernatant was transferred into a new microcentrifuge tube placed on ice. The supernatants were then incubated with a primary USP10 (4 μl) antibody overnight at 4 °C. Then the lysates were washed with RIPA buffer 3 times and the supernatant discarded. The samples were then boiled in 40 μl 1×loading buffer for 10 minutes and analyzed by immunoblotting. Protein samples were collected in-gel about 1 cm × 1 cm in size and analyzed by LC-MS.

### Cell viability assay

Primary rat hippocampal neurons were seeded in poly-D-lysine-coated 96-well plates and cultured for 7 days. A total of 4–6 replicate wells were set up for each group, and half of the maintenance medium was changed twice a week. Then the medium was replaced with 100 μl, of the medium containing different concentration of peptides. After 12 h incubation in the incubator at 37 °C. The neurons were further treated with 2 μM Aβ_42_ oligomers for 24 h and then 10 μl CCK-8 solution (Cat: #C0005, TargetMol was added to each well. Plates were incubated at 37 °C for 1–4 h. And then the absorbance at 450 nm was measured with a microplate reader. Cell viability = [A(Drug + )-A(Black)]/[A(Drug)-(A(Black)).

### Dataset

GSE5281 was downloaded from the Gene Expression Omnibus (GEO) database. The GSE5281 database collected information from six different brain regions of normal aged and AD brains with 161 samples, among which we chose samples of hippocampus including 13 controls and 10 AD patients. We used RStudio for the data analysis.

### Statistical analysis

Experimental values were obtained from at least three independent experiments with a similar pattern. Data were expressed as mean ± SEM and analyzed using GraphPad Prism 9.0 software. The level of significance between two groups was assessed with an unpaired t-test with Welch’s correction, while between more than two groups, one-way ANOVA and Bonferroni’s multiple comparison test were applied. Differences with *p* < 0.05 were considered as significant.

## Supplementary information

Supplementary Fig.1

Supplementary Fig.2

Supplementary Fig.3

Supplementary Fig.4

Supplementary Fig.5

Supplmentary legends

Supplementary Table S1

Supplementary Table S2

Supplementary Table S3

reproducibilty checklist

Original Western blots

## Data Availability

The data are available to academic researchers upon request.
