# Supplementary figures and images for: USP10 deubiquitinates Tau, mediating its aggregation
Source: Cell Death Dis. 2022 Aug 20;13(8):726. doi: 10.1038/s41419-022-05170-4 (PMC9392799; doi:10.1038/s41419-022-05170-4)

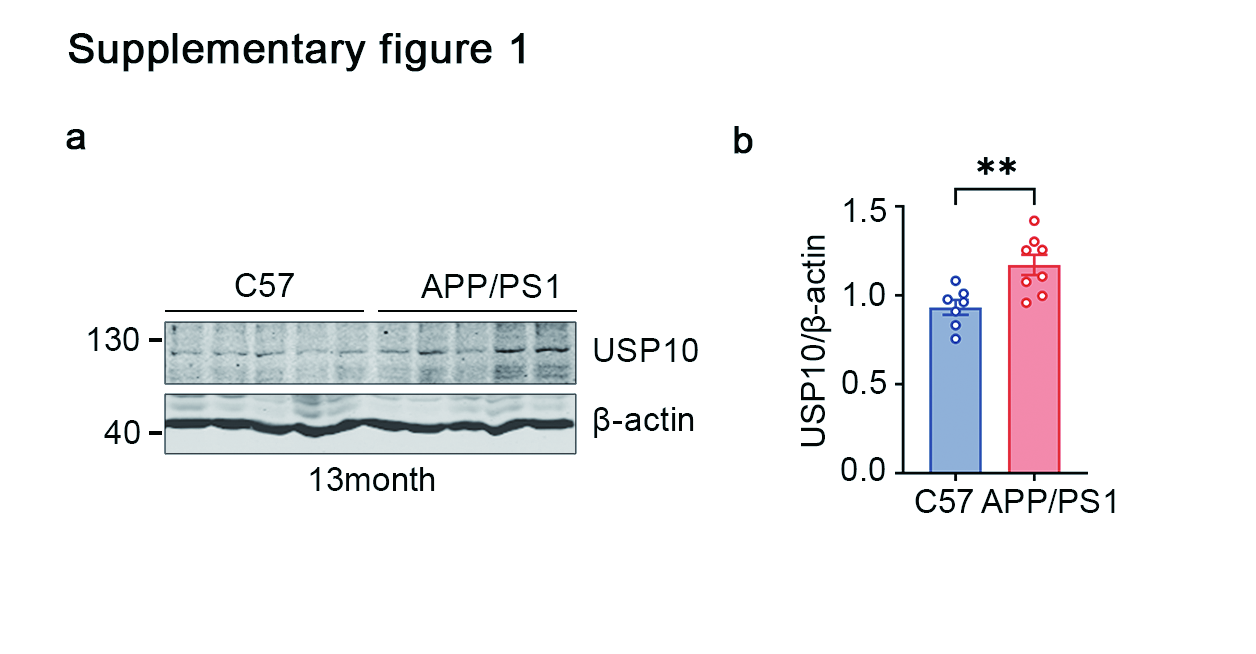

Supplement: Supplementary file 1 — Supplementary Fig.1 [file 41419_2022_5170_MOESM1_ESM.tif]

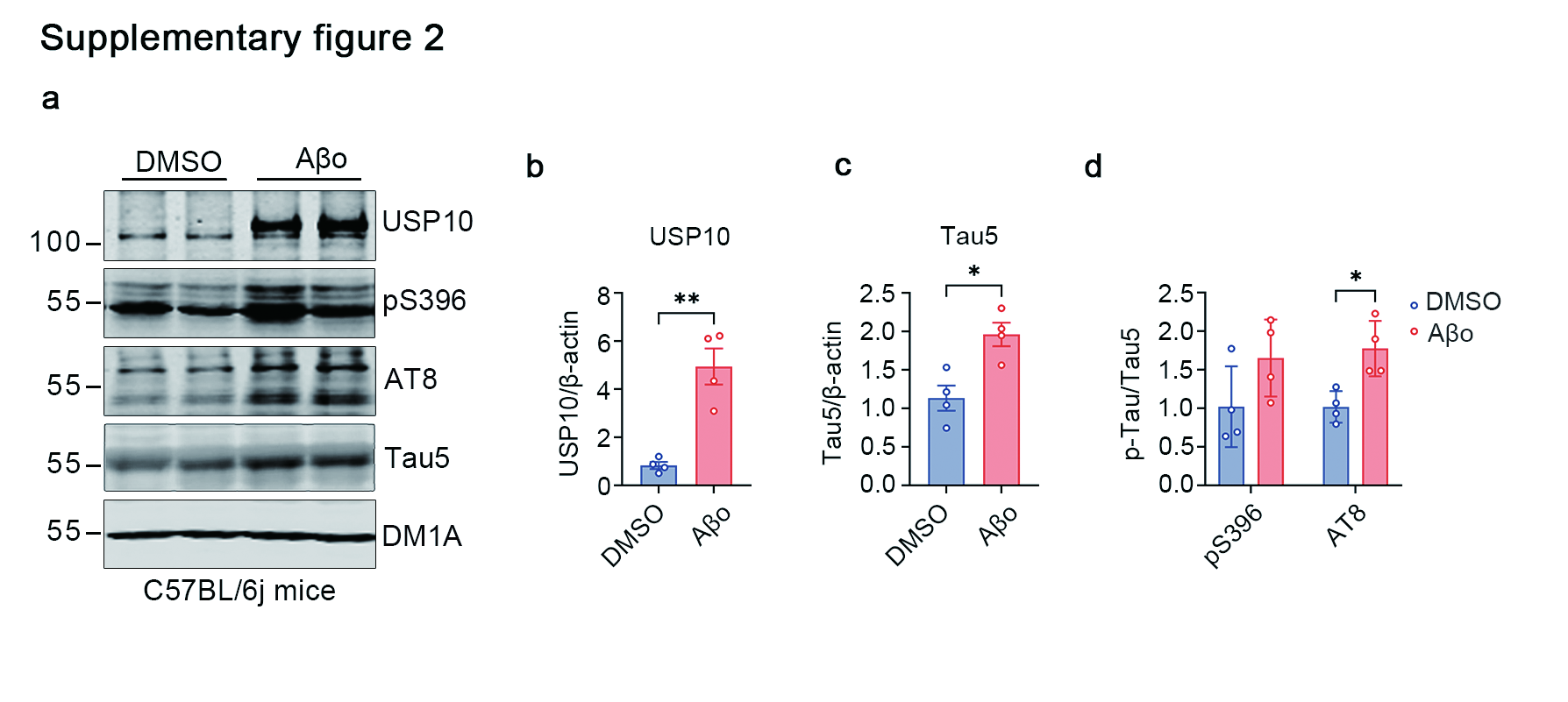

Supplement: Supplementary file 2 — Supplementary Fig.2 [file 41419_2022_5170_MOESM2_ESM.tif]

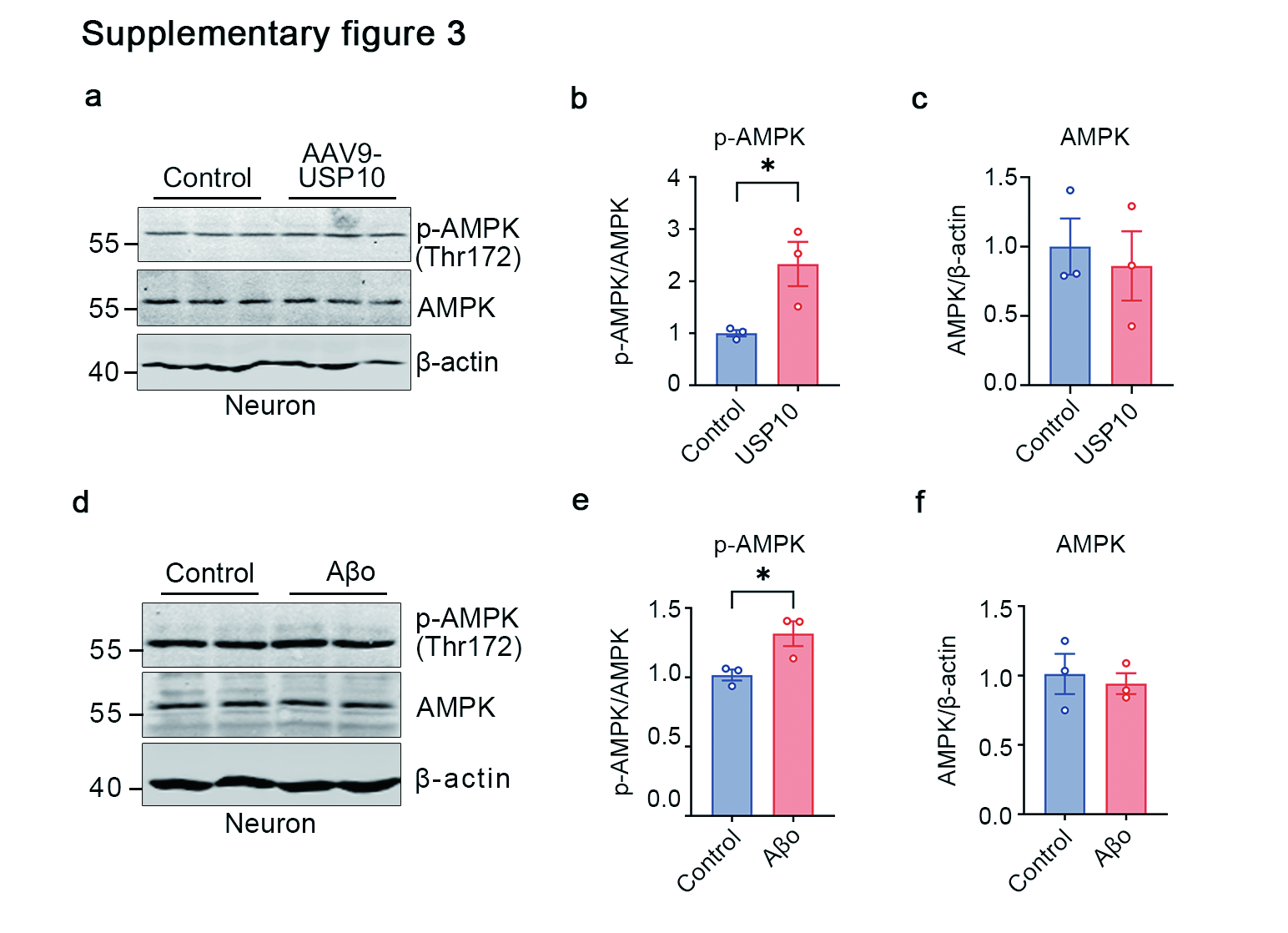

Supplement: Supplementary file 3 — Supplementary Fig.3 [file 41419_2022_5170_MOESM3_ESM.tif]

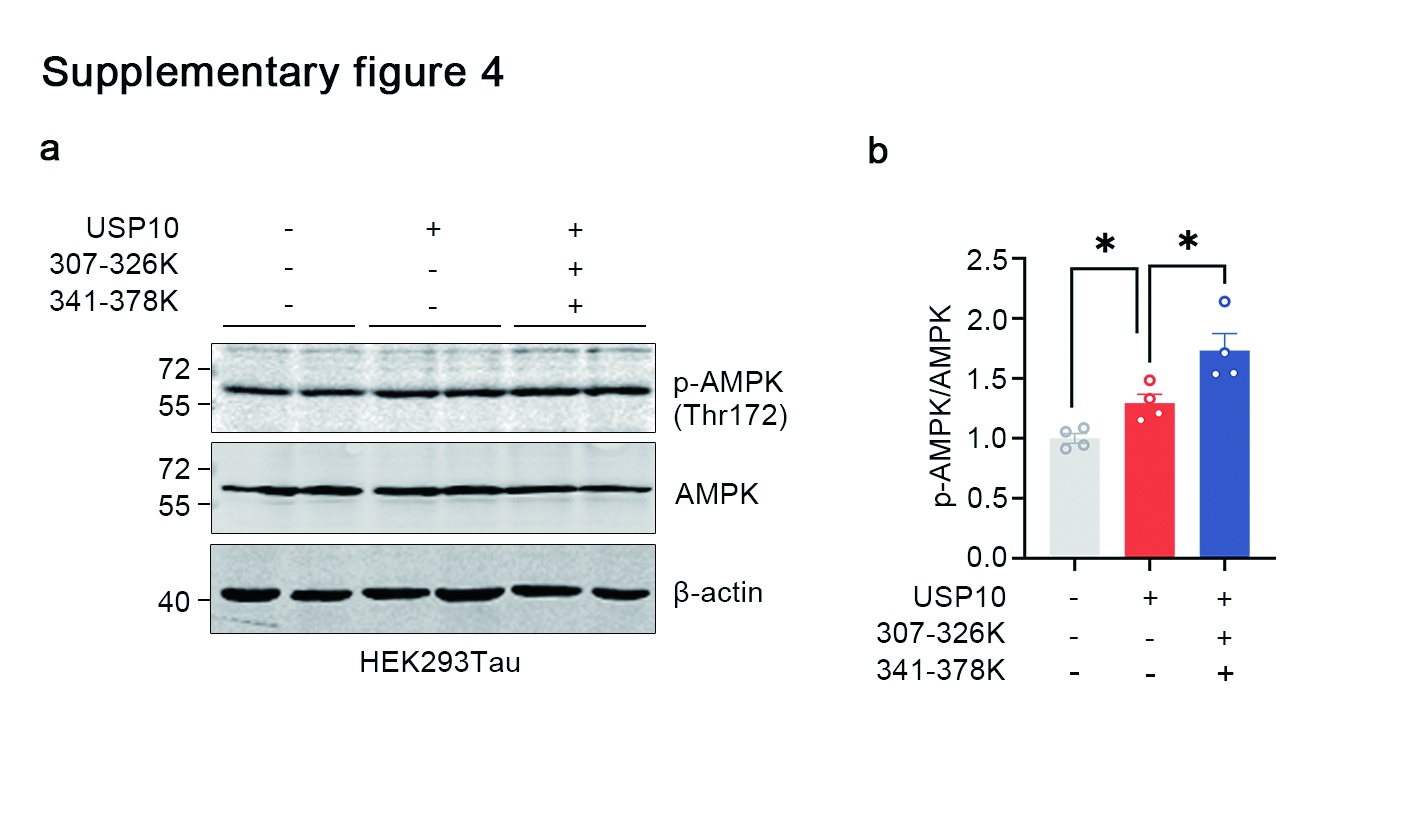

Supplement: Supplementary file 4 — Supplementary Fig.4 [file 41419_2022_5170_MOESM4_ESM.tif]

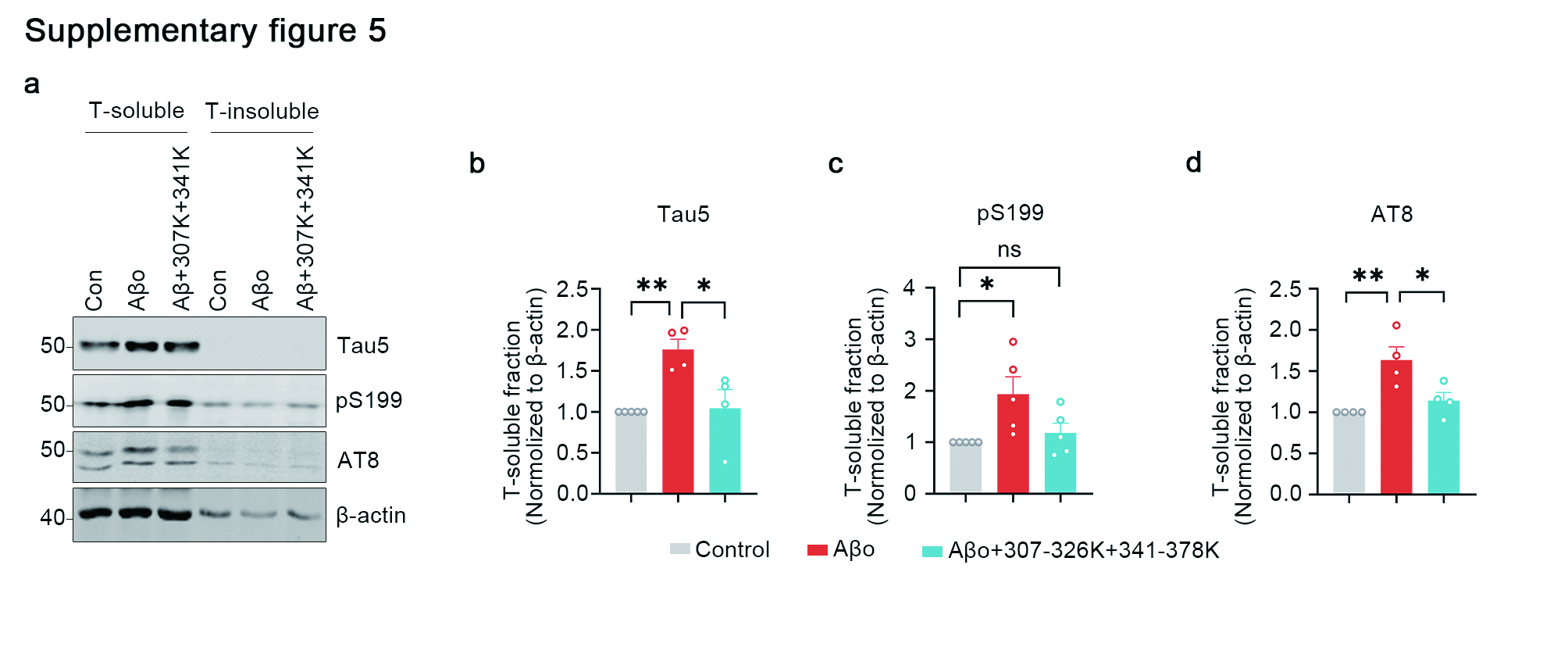

Supplement: Supplementary file 5 — Supplementary Fig.5 [file 41419_2022_5170_MOESM5_ESM.tif]

Original western blots


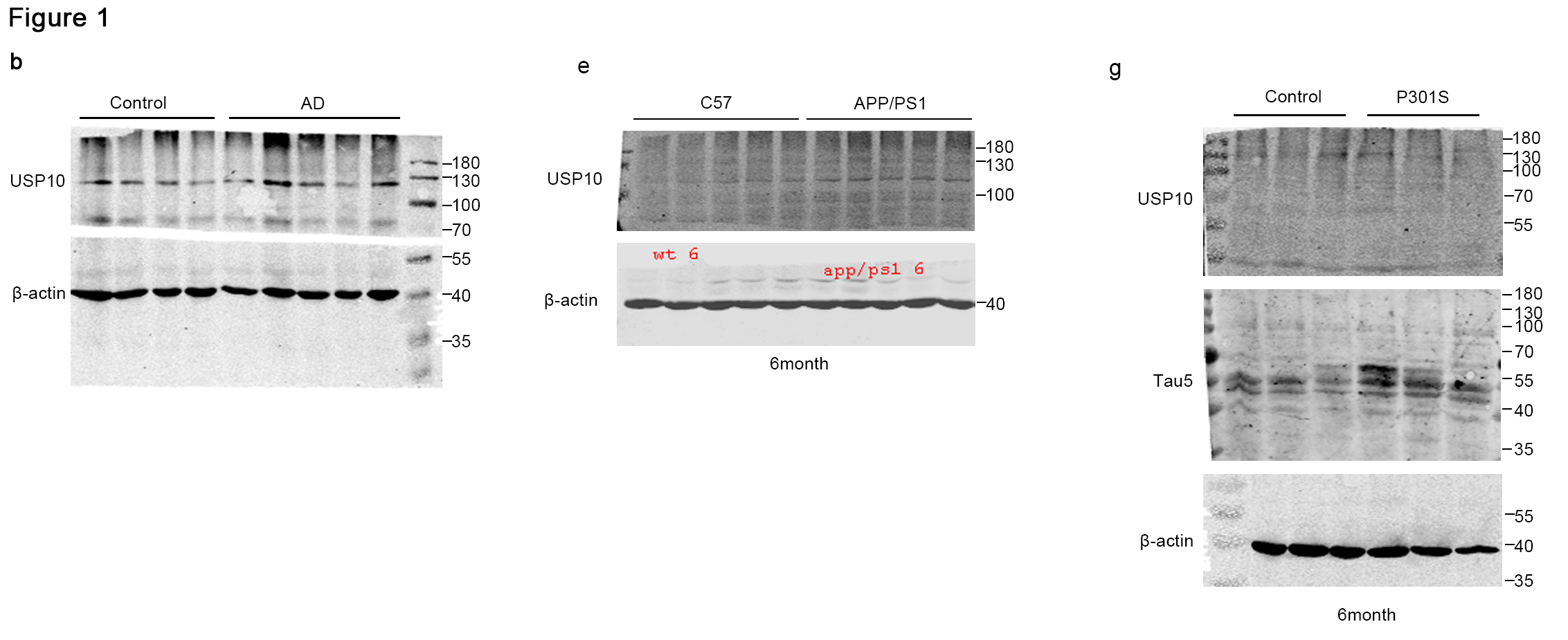


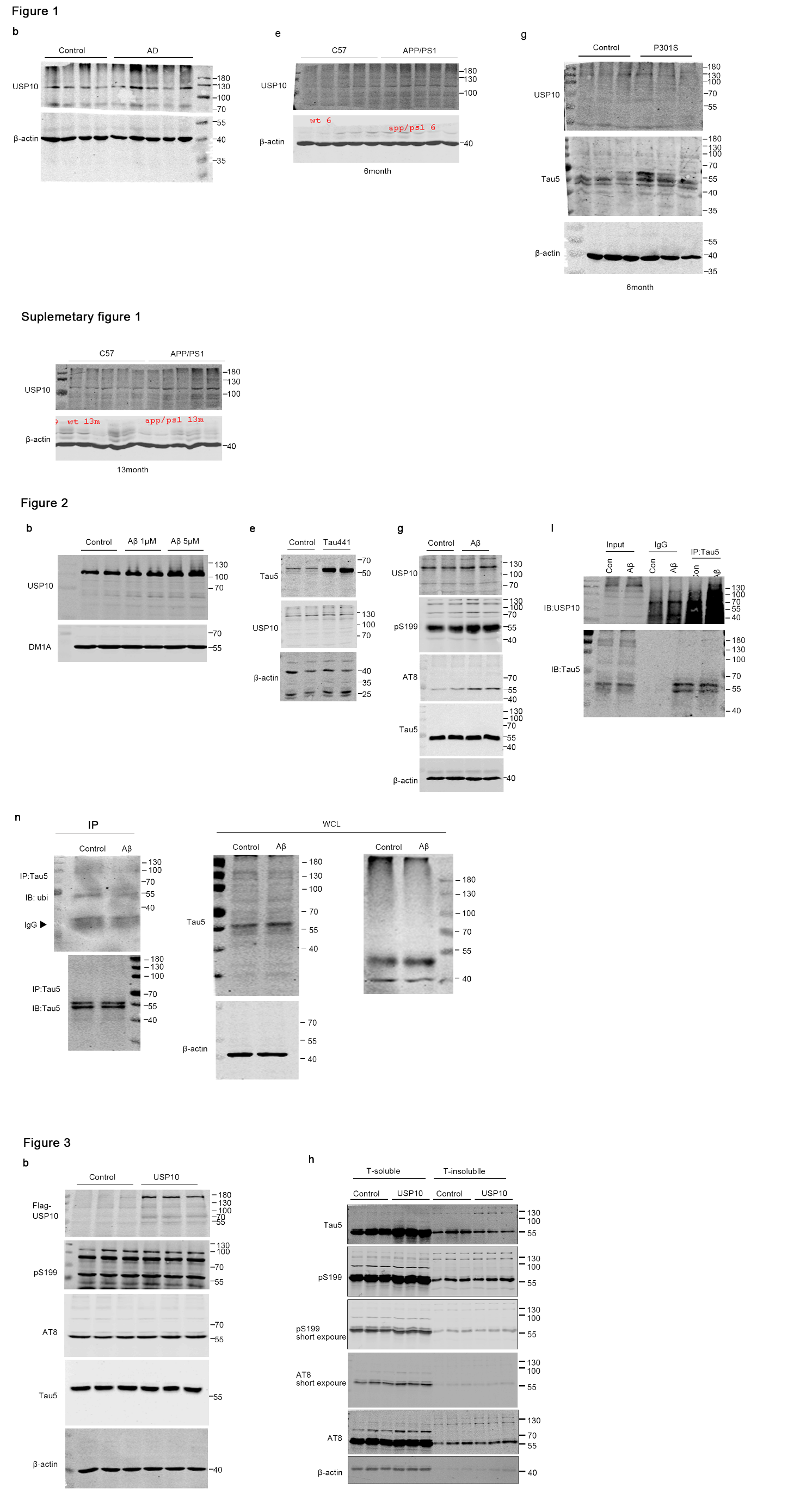


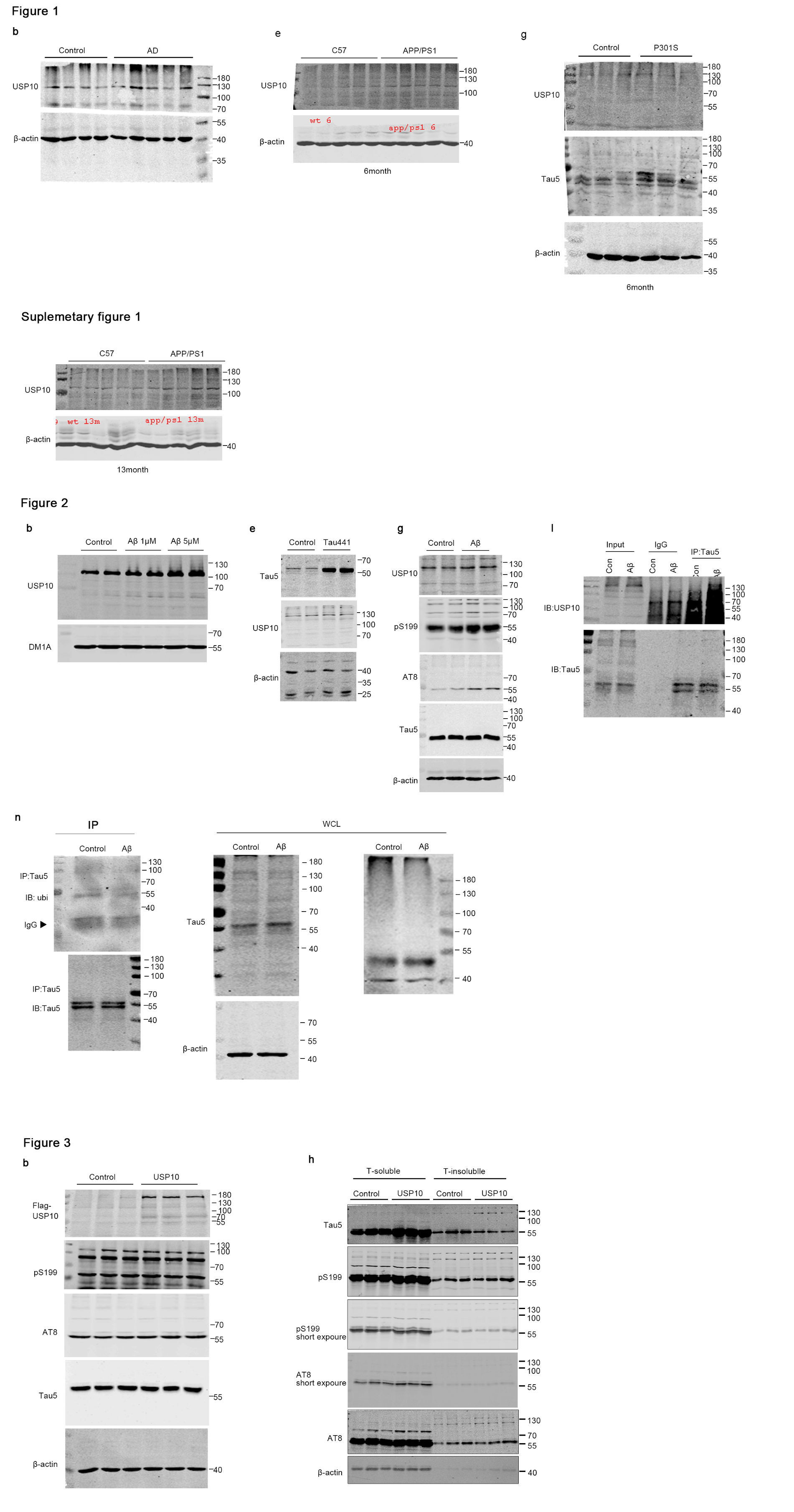


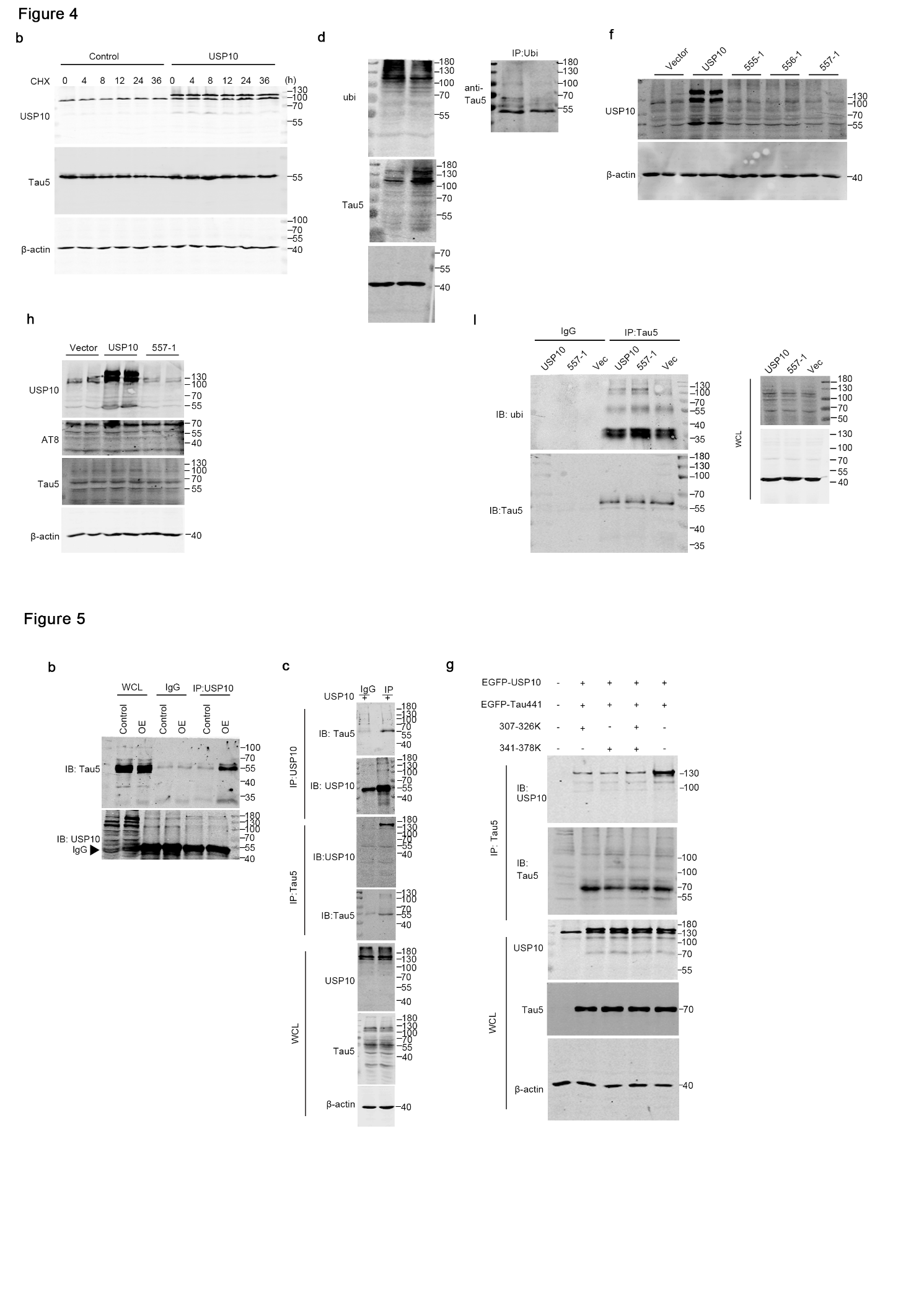


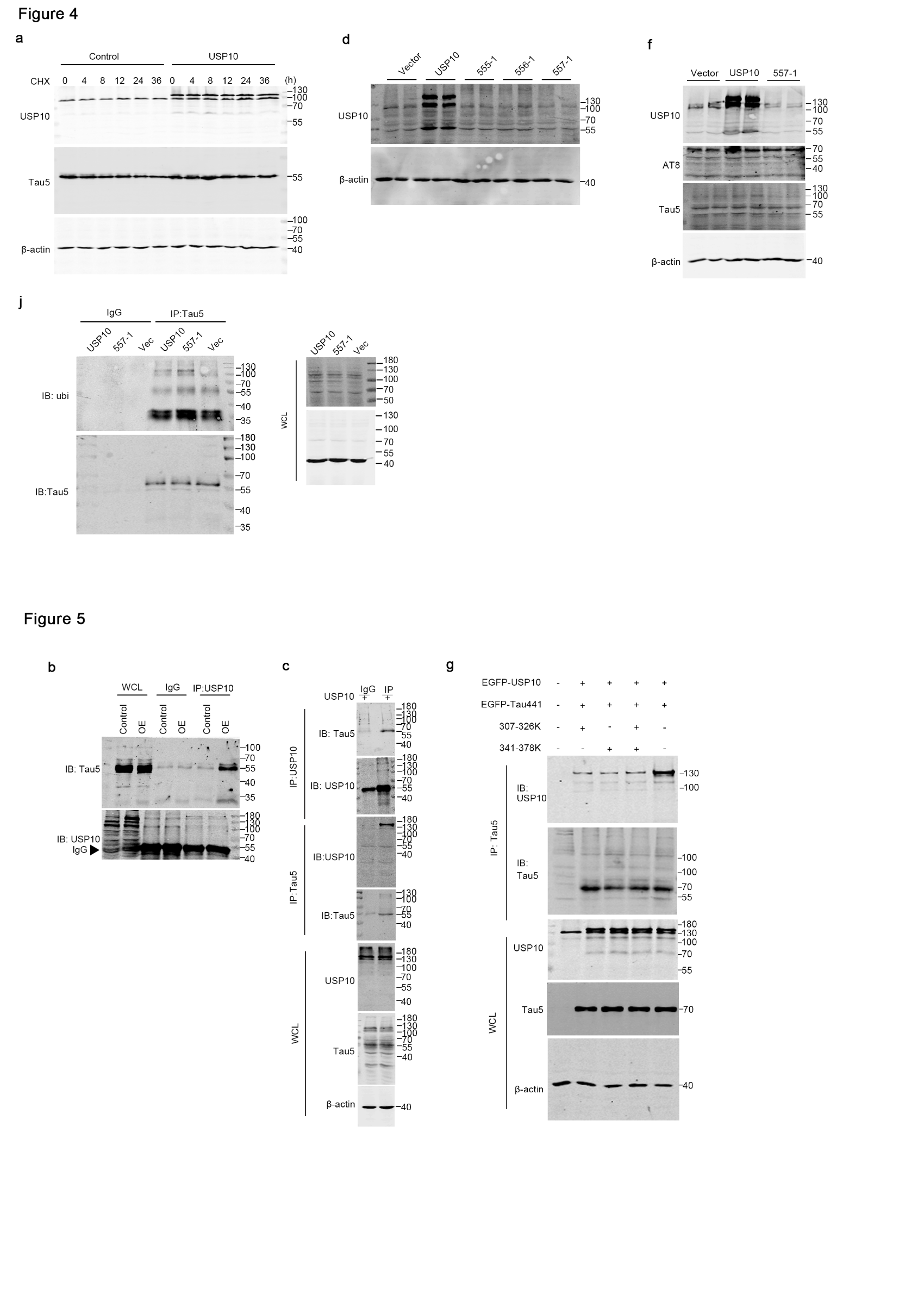


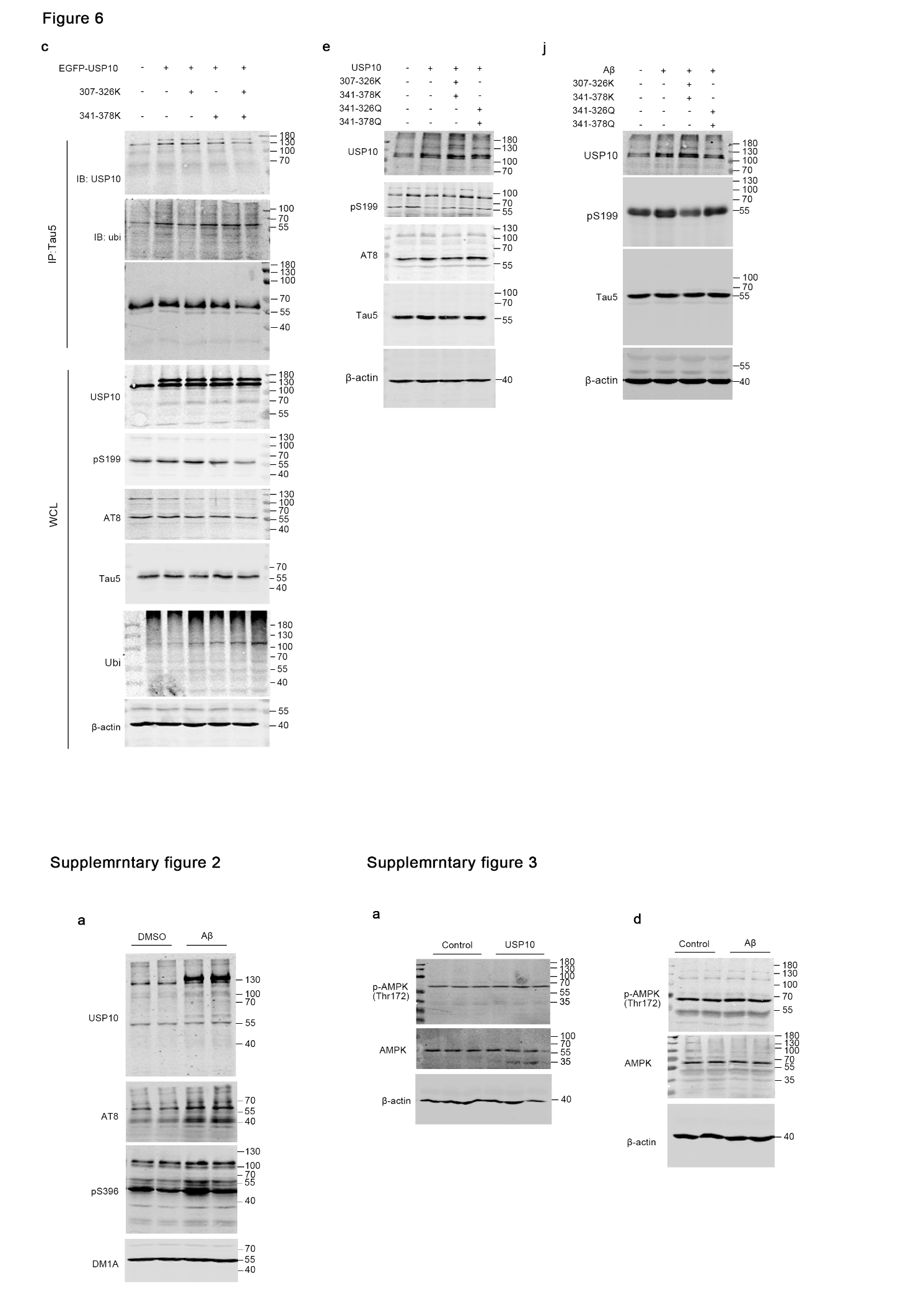


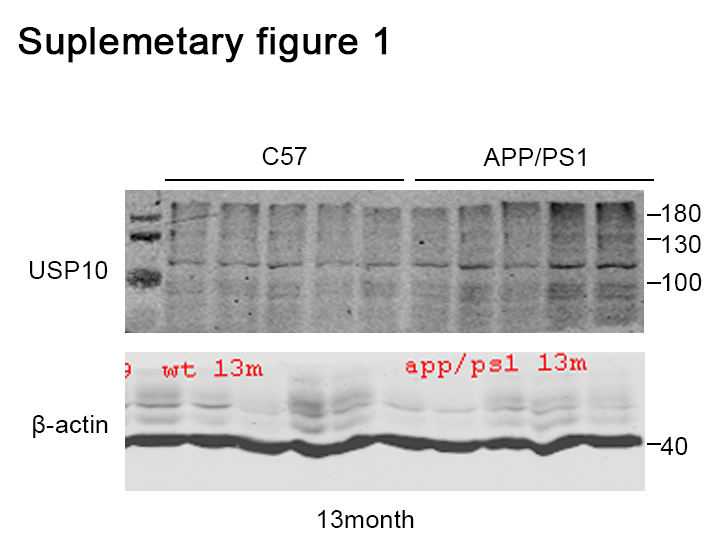

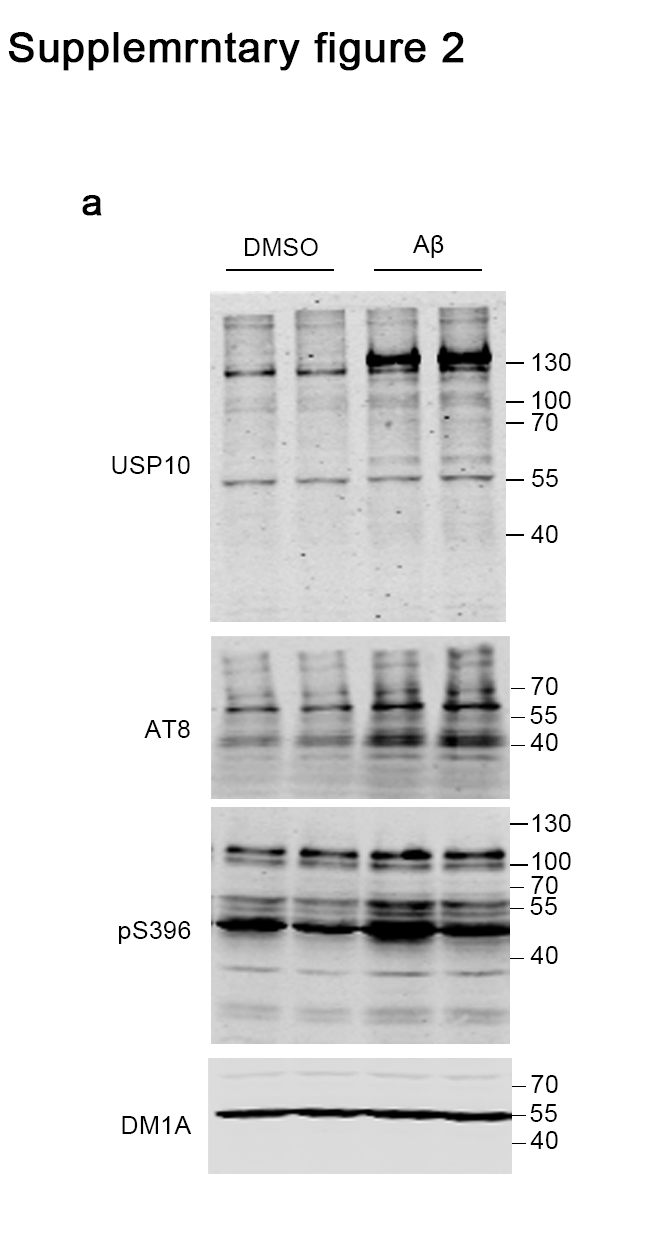


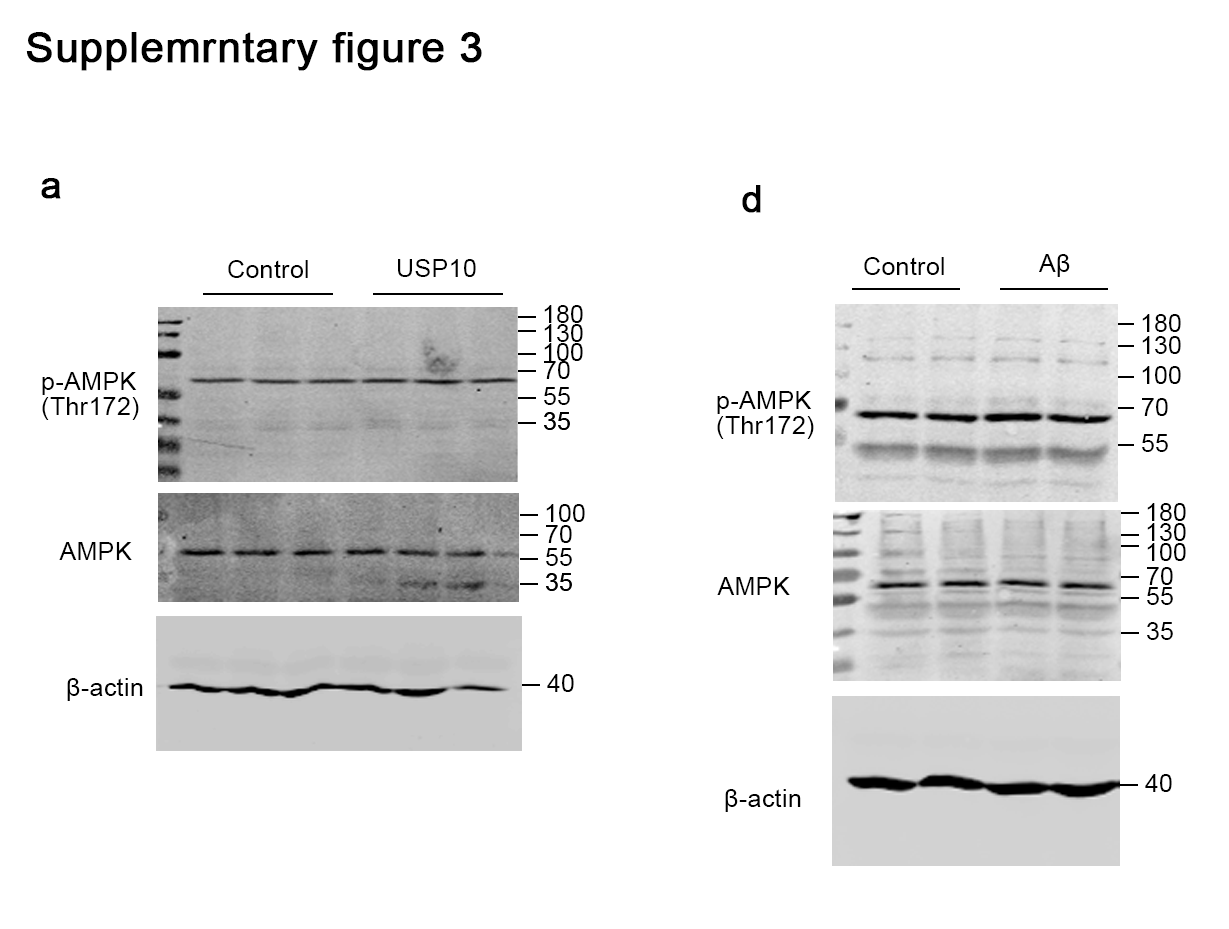


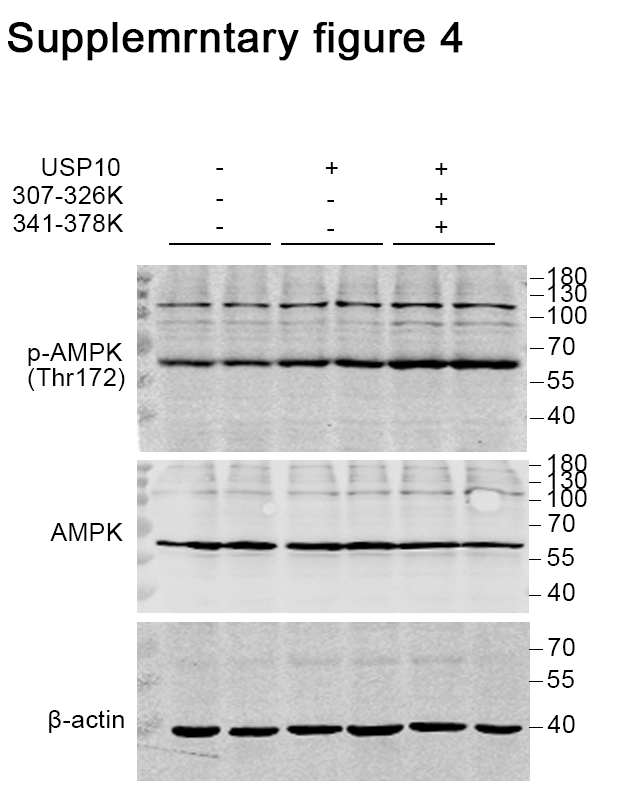

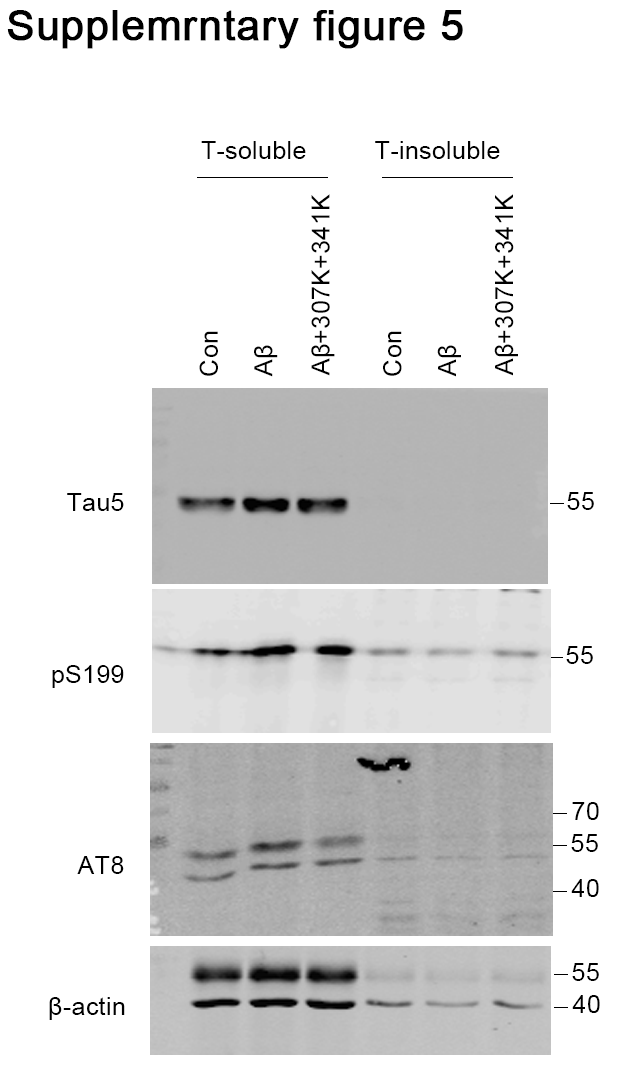

Supplement: Supplementary file 11 — Original Western blots [file 41419_2022_5170_MOESM11_ESM.docx]
