# Supplementary material for: USP10 deubiquitinates Tau, mediating its aggregation
Source: Cell Death Dis. 2022 Aug 20;13(8):726. doi: 10.1038/s41419-022-05170-4 (PMC9392799; doi:10.1038/s41419-022-05170-4)
Supplement: Supplementary file 6 — Supplmentary legends [file 41419_2022_5170_MOESM6_ESM.docx]

**Supplementary information**

**Supplementary Figure Legends**

**Supplementary Figure 1. USP10 is significantly increased in 13-month-old APP/PS1 mice.**

**a**. USP10 was detected in the hippocampus of 13-month-old APP/PS1 mice. n=7 C57BL/6j, n=9 APP/PS1 mice. All data represent mean ± SEM, p-value significance is calculated from a two-tailed t-test compared with control, **p < 0.01.

**Supplementary Figure 2. Aβo promotes USP10, and both total and hyperphosphorylated Tau upregulation *in vivo*.**

**a-d.** The injection of Aβo for 1 week in the lateral ventricles of wild-type C57BL/6j mice, hippocampi samples were extracted and examined by WB. The panels showed immunoblots of USP10, pS396, AT8, and Tau5, and p-Tau was normalized by Tau5. All data represent mean ± SEM, p-value significance is calculated from a two-tailed t-test compared with control, *p < 0.05; **p < 0.01

**Supplementary Figure 3. AMPK activation was induced by USP10 overexpression or Aβ oligomer treatment.**

**a-c.** Primary neurons were infected with AAV9-USP10 on DIV4. The level of p-AMPK(T172) was normalized by AMPK, that of AMPK by β-actin. **d-f**. The treatment of 2μM Aβ_42_ oligomers in primary neurons, and the graph showing the level of p-AMPK(Thr172) and AMPK. p-AMPK normalized by AMPK and AMPK was normalized by β-actin; n=3 independent experiments. All data represent mean ± SEM, p-value significance is calculated from a two-tailed t-test compared with control, *p < 0.05.

**Supplementary Fig 4. Interfering peptides have no influence on the activation of AMPK by expressing USP10.**

**a.** HEK293Tau cells overexpressing full-length USP10 with the vehicle were treated with 50μM of Tau 307-326K + Tau341-378K for 48h. **b.** Graph showing the level of p-AMPK (Thr172) normalized by AMPK; n=3 independent experiments. All data represent mean ± SEM, p-value significance is calculated from a one-way ANOVA, *p < 0.05.

**Supplementary Figure 5. Interfering peptides abrogate the Aβ_42_ oligomers-induced increased soluble Tau in primary neurons.**

**a.** Rat primary neurons were treated as mentioned in Fig. 6j. Soluble and insoluble proteins were extracted as indicated, and then subjected to WB. **b-d**. Graph showing the level of Tau5, pS199, and AT8; n=4 independent experiments. All data represent mean ± SEM, p-value significance is calculated from a one-way ANOVA, *p < 0.05; **p < 0.01; ns, not significant.
