# Supplementary material for: USP10 deubiquitinates Tau, mediating its aggregation
Source: Cell Death Dis. 2022 Aug 20;13(8):726. doi: 10.1038/s41419-022-05170-4 (PMC9392799; doi:10.1038/s41419-022-05170-4)
Supplement: Supplementary file 7 — Supplementary Table S1 [file 41419_2022_5170_MOESM7_ESM.docx]

**Table 1 Information of human samples:**

| Sample | Age | Sex | Brain region | Braak stage |
| --- | --- | --- | --- | --- |
| Control-1 | 72 | Male | Hippocampus | N/A |
| Control-2 | 74 | Male | Hippocampus | N/A |
| Control-3 | 83 | Female | Hippocampus | N/A |
| Control-4 | 86 | Male | Hippocampus | N/A |
| Control-5 | 98 | Male | Hippocampus | N/A |
| Control-6 | 70 | Female | Hippocampus | N/A |
| AD-1 | 83 | Male | Hippocampus | 4 |
| AD-2 | 78 | Male | Hippocampus | 4 |
| AD-3 | 71 | Female | Hippocampus | 4 |
| AD-4 | 73 | Male | Hippocampus | 4 |
| AD-5 | 86 | Female | Hippocampus | 6 |
| AD-6 | 79 | Male | Hippocampus | 6 |
| AD-7 | 99 | female | Hippocampus | 5 |
