# Supplementary material for: USP10 deubiquitinates Tau, mediating its aggregation
Source: Cell Death Dis. 2022 Aug 20;13(8):726. doi: 10.1038/s41419-022-05170-4 (PMC9392799; doi:10.1038/s41419-022-05170-4)
Supplement: Supplementary file 8 — Supplementary Table S2 [file 41419_2022_5170_MOESM8_ESM.docx]

**Table 2. Antibodies and reagents employed in this study**

| **ANTIBODY/REAGENT** | **TYPE** | **DILUTION** | **SOURCE** |
| --- | --- | --- | --- |
| AT8 | mAb | 1:1000 for WB  1:200 for IHC | Invitrogen, #MN1020 |
| pS199 | pAb | 1:1000 for WB | ZEN BIOSCIENCE, #382945 |
| pS396 | pAb | 1:1000 for WB | SAB, #11102 |
| Tau5 | mAb | 1:1000 for WB  1:200 for IF  1:100 for IP | Abcam, ab80679 |
| Tau5 | pAb | 1:1000 for WB | ZEN BIOSCIENCE, #R22821 |
| ubiquitin | pAb | 1:1000 for WB  1:100 for IP | Cell Signaling Technology, #3933S |
| USP10 | pAb | 1:1000 for WB  1:200 for IF | Abcam, #Ab70895 |
| Flag | mAb | 1:1000 for WB | Sigma-Aldrich, #F1804 |
| AMPKα_1_/ AMPKα_2_ | pAb | 1:1000 for WB | ABclonal Biotechnology Co.,Ltd , #A12718 |
| p-AMPKα(Thr172) | pAb | 1:1000 for WB | Cell Signaling Technology, #50081S |
| β-actin | pAb | 1:3000 for WB | ABclonal Biotechnology Co.,Ltd, #AC026 |
| DM1A | mAb | 1:1000 for WB | Sigma-Aldrich, #T9026 |
| Alexa Fluor® 488 AffiniPure donkey anti- Mouse IgG | pAb | 1:200 for IF | Jackson Immuno Research labs, #715-546-151 |
| Alexa Fluor® 594 AffiniPure Donkey Anti-  Rabbit IgG | pAb | 1:200 for IF | Jackson Immuno Research labs, #711-585-152 |
| Antifade Mounting Medium with DAPI | N/A | N/A | Beyotime Biotechnology, #P0131 |
| IRDye® 800CW Goat anti-Rabbit IgG Secondary Antibody | pAb | 1:10000 for WB | LI-COR Biosciences, #926-32211 |
| IRDye® 800CW Goat anti-Mouse IgG Secondary Antibody | mAb | 1:10000 for WB | LI-COR Biosciences, #926-32210 |
| Protein ladders | N/A | N/A | Thermo Fisher, #26617 |
| Mouse IgG Two-step IHC Detection reagent kit | N/A | N/A | Boster Biological Technology CO.,Ltd, #SV0001 |
| DAB Substrate Kit (20×) | N/A | N/A | Zsbio Commerce Store, Beijing, China, #ZLI-9019 |
| Cycloheximide | N/A | N/A | MCE, HY-12320 |
| Protein A+G magnetic Beads | N/A | N/A | Biolinkedin, #L-1004 |
| RIPA lysis buffer (weak) | N/A | N/A | Beyotime Biotechnology, #P0013D |
| RIPA lysis buffer (strong) | N/A | N/A | Beyotime Biotechnology, #P0013B |
| HighGene transfect reagent | N/A | N/A | ABclonal Biotechnology Co.,Ltd, #RM09014 |

IHC, immunohistochemistry; IF, immunofluorescence; WB, western blotting; IgG, immunoglobulin G; mAb, monoclonal antibody; pAb, polyclonal antibody
